# Synthesis, characterization and luminescence studies of gold(I)–NHC amide complexes

**DOI:** 10.3762/bjoc.9.260

**Published:** 2013-10-28

**Authors:** Adrián Gómez-Suárez, David J Nelson, David G Thompson, David B Cordes, Duncan Graham, Alexandra M Z Slawin, Steven P Nolan

**Affiliations:** 1 EaStCHEM School of Chemistry, University of St Andrews, North Haugh, St Andrews, Fife, KY16 9ST, UK; 2 WestCHEM Department of Pure and Applied Chemistry and Centre for Molecular Nanometrology, University of Strathclyde, 295 Cathedral Street, Glasgow, G1 1XL, UK

**Keywords:** fluorescence, gold, gold catalysis, *N*-heterocyclic carbenes, synthesis

## Abstract

A flexible, efficient and straightforward methodology for the synthesis of *N*-heterocyclic carbene gold(I)–amide complexes is reported. Reaction of the versatile building block [Au(OH)(IPr)] (**1**) (IPr = 1,3-bis(2,6-diisopropylphenyl)imidazol-2-ylidene) with a series of commercially available (hetero)aromatic amines leads to the synthesis of several [Au(NRR’)(IPr)] complexes in good yields and with water as the sole byproduct. Interestingly, these complexes present luminescence properties. UV–vis and fluorescence measurements have allowed the identification of their excitation and emission wavelengths (λ_max_). These studies revealed that by selecting the appropriate amine ligand the emission can be easily tuned to achieve a variety of colors, from violet to green.

## Introduction

**Scheme 1:**
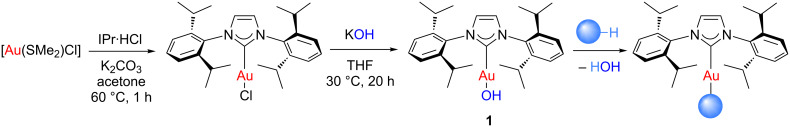
Straightforward synthesis of organogold complexes via deprotonation reactions, using **1**.

The synthesis of organogold complexes has recently attracted wide attention due to their considerable range of applications, in areas such as materials and medicinal chemistry, as well as their potential role as reaction intermediates or new catalysts in gold-catalyzed processes [1–7]. This has led several research groups to investigate general, green and straightforward methodologies for the synthesis of organogold complexes. We have focused on the synthesis and study of transition metal complexes bearing *N*-heterocyclic carbene (NHC) ligands [8–10]. Recently, we have been very active in the synthesis and characterization of new gold(I)–NHC complexes and the study of their reactivity, with a special focus on the development of straightforward methodologies [11–12]. As a result of our investigations, we have recently reported the synthesis of [AuX(NHC)] (X = Cl, Br, I) complexes, directly from imidazolium and imidazolidinium salts and a suitable gold source, such as [AuCl(SMe_2_)], using K_2_CO_3_ as a base [13]. Moreover, we have also reported the synthesis of the first mononuclear gold(I) hydroxide species, [Au(OH)(IPr)] (**1**) (IPr = 1,3-bis(2,6-diisopropylphenyl)imidazol-2-ylidene), using [AuCl(IPr)] and an excess of KOH in THF [14–15]. This complex has proven to be an excellent synthon for the preparation of a wide variety of organogold(I) species [16–23]. Two approaches have been developed for the synthesis of such species using hydroxide **1**: a) via transmetallation from either boronic acids or siloxides, or b) via deprotonation reactions of suitable substrates. We have applied the latter methodology to the synthesis of organogold–NHC complexes bearing several *trans*-ligands, such as acetylene, phenyl and phenol derivatives. These reactions can be carried out using reagent-grade materials, without the requirement to exclude light, air or moisture; providing an easy, straightforward 3-step route for the synthesis of organogold–NHC complexes from readily available gold sources, such as [Au(SMe_2_)Cl] (Scheme 1).

Toste and Bergman have recently reported the synthesis, characterization and reactivity studies of a series of [Au(NRR’)(NHC)] complexes [24]. This study represents the first reported synthesis of gold(I)–NHC amide complexes. The procedure employed for the synthesis of these species required the use of lithium amide reagents that are not stable towards air or moisture [24]. Therefore, the development of a more robust approach would be desirable. In addition to the interesting reactivity shown by Toste and Bergman, Hoffman and Viseux have probed the anticancer properties of gold amide complexes, reacting modified triflamide compounds with phosphine-bearing gold chloride species [25]. Taking into account the interest in these type of complexes, we sought to use hydroxide **1** to develop an easy, green (in such deprotonation reactions, water is the sole byproduct) and straightforward methodology for the synthesis of gold(I)–amide complexes.

## Results and Discussion

### Synthesis and characterization of gold(I)–amide complexes

We began our studies by exposing hydroxide **1** to a series of alkyl- and arylamines. While no reaction was observed with either morpholine or isopropylamine, the use of 1 equiv of aniline or 2-aminopyridine led to the isolation of complexes **2** and **3** respectively, in good yields after overnight reaction at room temperature. These results are consistent with the known p*K*_a_ of [Au(OH)(IPr)] (30.3) [26]; the scope of this preparative route is, as with other deprotonation reactions with this building block, limited to substrates with a p*K*_a_ lower than 30.

**Scheme 2:**
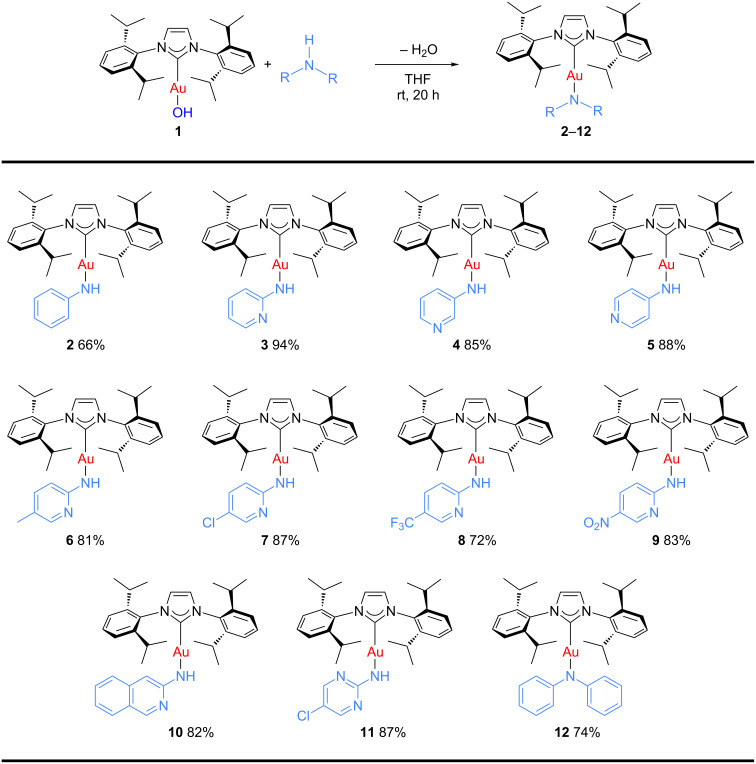
Scope of the reaction between **1** and several (hetero)aromatic amines. Reaction conditions: **1** (1 equiv), amine (1 equiv), THF (0.5–2 mL), rt, 20 h.

Encouraged by these exploratory reactions, a series of (hetero)aromatic amines were employed to prepare the corresponding gold(I)–amide complexes. The desired complexes were obtained by reaction of [Au(OH)(IPr)] (**1**) with each (hetero)aromatic amine in THF at room temperature for 20 h. A range of aromatic amines were employed, including aniline, diphenylamine, pyridines, a pyrimidine and one isoquinoline. The corresponding complexes were obtained in analytically pure form and in good yields as yellow or white microcrystalline powders after a simple work-up (Scheme 2). All complexes were characterized by ^1^H and ^13^C{^1^H} NMR spectroscopy and elemental analysis (see Supporting Information File 1). The new species are bench stable in the solid state, and do not require special handling. However, some do decompose slowly in solution over the course of a number of hours.

**Figure 1:**
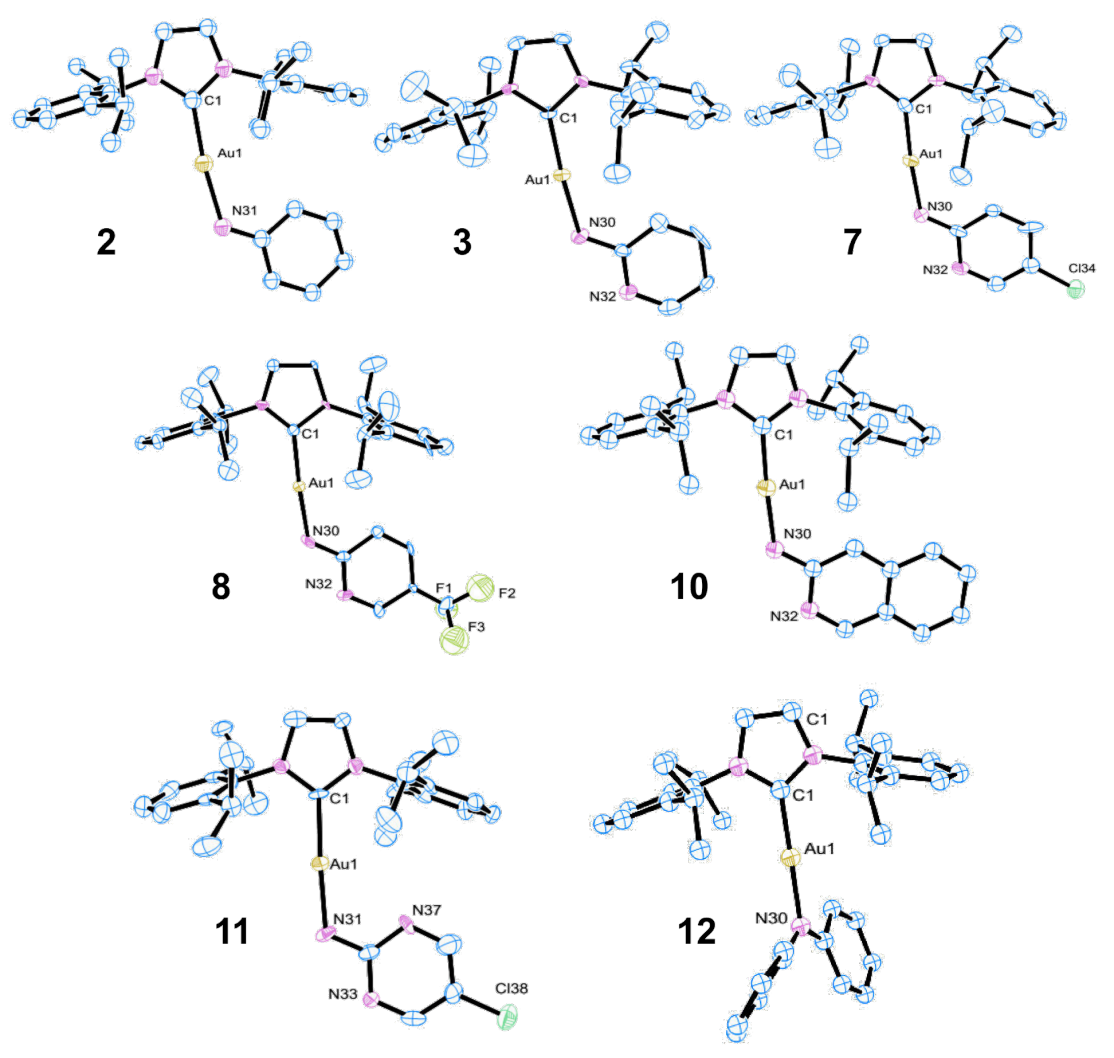
X-ray crystal structures of complexes **2**, **3**, **7**, **8**, **10**, **11** and **12**. Hydrogen atoms are omitted for clarity.

A selected number of these complexes were characterized by X-ray crystallography, as further support to confirm atom connectivity and molecular geometry (Figure 1) [27].

All complexes were found to display the expected two-coordinate linear geometry around the metal center, with all C–Au–N angles in the range 173–179°. There was no evidence of any interactions between the gold center and the heteroatoms in complexes **3**, **7**, **8**, **10** or **11**. However, for **3**, **7**, **8**, **10** and **11** there is an intermolecular contact between the aromatic nitrogen atom and the NHC ligand backbone proton (d_N-H_ = 2.27 Å–2.34 Å). All gold–carbon bond lengths were in the range 1.95 Å (**8**) to 2.02 Å (**2**), while gold–nitrogen bond lengths varied from 1.98 Å (**8**) to 2.06 Å (**2**).

**Figure 2:**
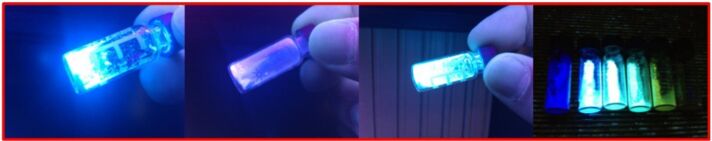
Selected examples of gold–NHC amide complexes under UV light (λ = 366 nm).

During the characterization of the gold–NHC amide complexes we observed that some of the complexes possessed luminescent properties when exposed to ultraviolet light (λ = 366 nm) in the solid state (Figure 2).

A number of luminescent organogold complexes have been reported in the literature, often demonstrating interesting properties that may lead to their application in materials science and the preparation of optical materials [28–34]. Amongst these, NHC-bearing gold complexes have been shown by several research groups to be very useful luminescent materials [19,35–39], and have attracted industrial interest [40–42]. Thus, we decided to explore the potential of these gold amide species as luminescent materials.

We began our luminescence studies by recording the UV–vis spectra of the aforementioned gold–amide complexes using a dilute (ca. 0.2 mmol/L) CH_2_Cl_2_ solution. The wavelengths of the absorption maxima on the UV–vis spectra were in the range of 250–350 nm for most complexes, with the exception of 2-amino-5-nitropyridine derived **9** and 3-aminoisoquinoline-derived **10**, which exhibited absorption maxima at ca. 430 nm (see Supporting Information File 1). These data are consistent with the physical appearance of complexes **2–12** in the solution state, i.e. complexes **9** and **10** are yellow.

**Figure 3:**
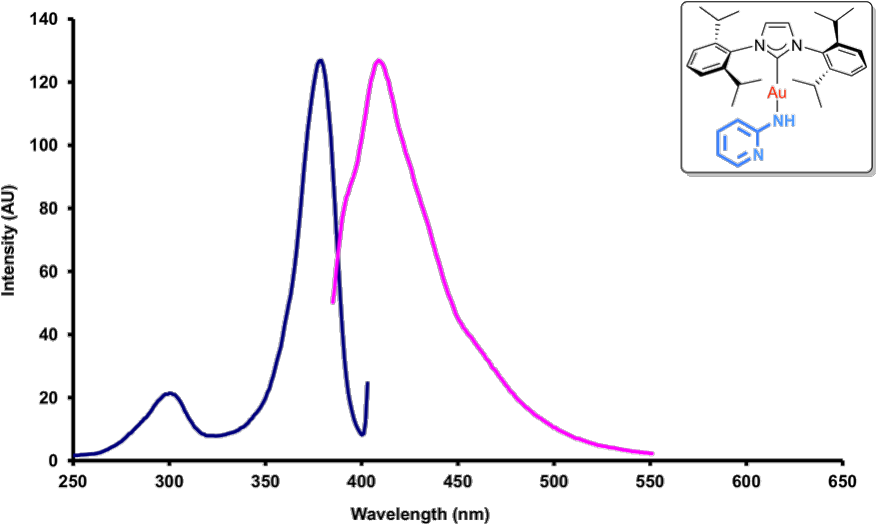
Excitation (blue) and emission (pink) data for complex **3**, bearing a 2-pyridine ligand (see inset).

The excitation and emission maxima were then determined. These measurements were conducted on more concentrated (ca. 4 mmol/L) CH_2_Cl_2_ solutions, compared to those used for UV–vis spectroscopy. In each case, the relevant maxima could be identified. An example dataset is presented in Figure 3. Lifetimes for all luminescence measurements were ≤1 μs (at the limit of the apparatus), suggesting that the luminescence was due to fluorescence, rather than phosphorescence.

The wavelengths of emission maxima spans the range 390 to 516 nm, demonstrating that by selecting the appropriate amine ligand the emission can be easily tuned to achieve a variety of colors, i.e. from violet (**3**, λ_max_ = 409 nm) to green (**4**, λ_max_ = 516 nm). In each case, there is a considerable Stokes shift (27 to 166 nm). In terms of the intensity of emission, a wide range of values were recorded, although complexes could be considered as part of one of three distinct groups. Phenyl-bearing complexes (such as **2** and **12**), 4-aminopyridine-derived **5** and electron-poor **9** showed relatively weak emission (<10 AU). Electron-poor 2-aminopyridine derivatives, 3-aminopyridine-derived **4** and 2-amino-5-chloropyrimidine-derived **11** exhibited moderate emission intensity (ca*.* 10–50 AU). The most intense fluorescence was observed with complexes **3**, **6** and **10**: 2-pyridine derivatives without electron-withdrawing substituents (ca. 120–290 AU) (Table 1).

**Table 1:** Fluorescence measurement data.^a^

entry	substituent	λ_max_/excitation (nm)	λ_max_/emission (nm)	intensity_max_ (AU)

**1**	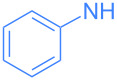 **2**	396	423	3.8
**2**	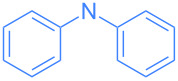 **12**	410	470	4.61
**3**	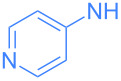 **5**	352	390	7.25
**4**	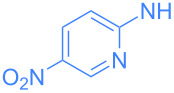 **9**	350	516	1.09
**5**	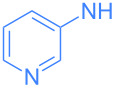 **4**	398	454	21.2
**6**	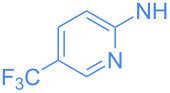 **8**	368	406	12.8
**7**	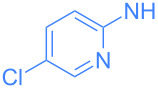 **7**	395	428	45.8
**8**	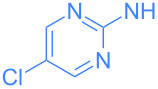 **11**	385	435	36.0
**9**	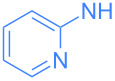 **3**	379	409	126
**10**	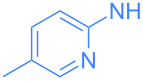 **6**	389	420	119
**11**	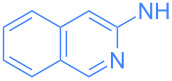 **10**	471	505	293

^a^At ca. 4 mmol/L in CH_2_Cl_2_ solution.

**Figure 4:**
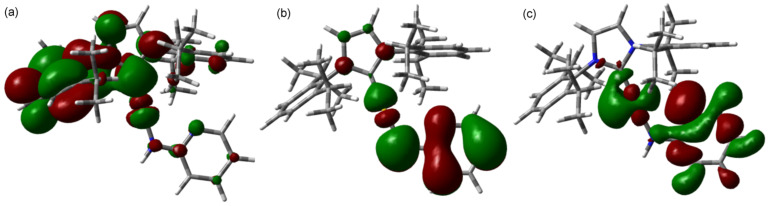
(a) LUMO, (b) HOMO and (c) HOMO-1 of complex **3**.

DFT calculations were used to probe the nature of the frontier orbitals of complex **3** (at the M06-L/SDD level of theory) [43] using Gaussian 09 [44]. The LUMO, HOMO and HOMO-1 are pictured in Figure 4. While the HOMO and HOMO-1 are centered predominantly on the amide ligand, the LUMO is localized on the aryl ring of the NHC ligand; we propose that the fluorescence behavior is due to a HOMO-to-LUMO transition. Therefore, the use of different NHC ligands should also allow access to different fluorescence behavior.

## Conclusion

A novel series of NHC-bearing gold(I)–amide complexes have been prepared using a simple, straightforward synthetic route that can be conducted using reagent-grade materials on the laboratory bench in air. The resulting species have been characterized using a number of methods, including NMR spectroscopy and X-ray crystal diffraction. These new species have been shown to be fluorescent, and their absorbance and emission maxima have been determined. Notably, there are key trends in the fluorescence behavior of these materials, with more electron-rich 2-pyridine derivatives showing strong emission, and isoquinoline-derived complexes showing the strongest fluorescence. While the present study has been conducted using commercial reagent-grade amines, there is significant scope to prepare a much wider range of gold(I)–amide complexes, including those prepared using designed aromatic amines. Further work is underway in our group to explore both the potential of gold(I)–NHC amide complexes, and further applications of gold(I) hydroxides as building blocks and catalysts.

## Supporting Information

File 1 Experimental part.
