# Supplementary material for: Synthesis, characterization and luminescence studies of gold(I)–NHC amide complexes
Source: Beilstein J Org Chem. 2013 Oct 28;9:2216–23. doi: 10.3762/bjoc.9.260 (PMC3817481; doi:10.3762/bjoc.9.260)
Supplement: File 1 — Experimental part. [file Beilstein_J_Org_Chem-09-2216-s001.pdf]

Supporting Information

for

Synthesis, characterization and luminescence studies of gold(I)–NHC amide complexes

Adrián Gómez-Suárez[1], David J. Nelson[1], David G. Thompson[2], David B. Cordes[1], Duncan Graham[2], Alexandra M. Z. Slawin[1] and Steven P. Nolan*[1]

Address: [1]EaStCHEM School of Chemistry, University of St Andrews, North Haugh, St Andrews, Fife, KY16 9ST, UK and [2]WestCHEM Department of Pure and Applied Chemistry and Centre for Molecular Nanometrology, University of Strathclyde, 295 Cathedral Street, Glasgow, G1 1XL, UK

Email: Steven P. Nolan – snolan@st-andrews.ac.uk

* Corresponding author

Experimental part

Table of Contents

General considerations: S3

Synthesis of (1,3-bis(2,6-diisopropylphenyl)imidazol-2-ylidene)(phenylamino)gold Complex (2): S3

Synthesis of (1,3-bis(2,6-diisopropylphenyl)imidazol-2-ylidene)(pyridin-2-ylamino)gold complex (3): S3

Synthesis of (1,3-bis(2,6-diisopropylphenyl)imidazol-2-ylidene)(pyridin-3-ylamino)gold Complex (4): S4

Synthesis of (1,3-bis(2,6-diisopropylphenyl)imidazol-2-ylidene)(pyridin-4-ylamino)gold complex (5): S4

Synthesis of (1,3-bis(2,6-diisopropylphenyl)imidazol-2-ylidene)((5-methylpyridin-2-yl)amino)gold Complex (6): S5

Synthesis of (1,3-bis(2,6-diisopropylphenyl)imidazol-2-ylidene)((5-chloropyridin-2-yl)amino)gold complex (7): S5

Synthesis of (1,3-bis(2,6-diisopropylphenyl)imidazol-2-ylidene)((5-(trifluoromethyl)pyridin-2-yl)amino)gold complex (8): S5

Synthesis of (1,3-bis(2,6-diisopropylphenyl)imidazol-2-ylidene)((5-nitropyridin-2-yl)amino)gold complex (9): S6

Synthesis of (1,3-bis(2,6-diisopropylphenyl)imidazol-2-ylidene)(isoquinolin-3-ylamino)gold complex (10): S6

Synthesis of (1,3-bis(2,6-diisopropylphenyl)imidazol-2-ylidene)((5-chloropyrimidin-2-yl)amino)gold complex (11): S7

Synthesis of (1,3-bis(2,6-diisopropylphenyl)imidazol-2-ylidene)(diphenylamino)gold complex (12): S7

UV–vis and fluorescence spectra: S8

NMR spectra S19

DFT Calculations S30

General considerations:

Unless otherwise stated, all solvents and reagents were used as purchased and all reactions were performed under air. Deuterated solvents (e.g. CD_2Cl_2) were filtered through basic alumina in order to remove traces of HCl. NMR spectra were recorded on 500, 400 and 300 MHz spectrometers at room temperature in CD_2Cl_2. Chemical shifts (δ) are reported in ppm, relative to the solvent peaks ($CDHCl_2$, 5.32 ppm for ^{1}H; CD_2Cl_2, 53.84 ppm for ^{13}C). Data for ^{1}H NMR are reported as follows: chemical shift, multiplicity (s = singlet, d = doublet, t = triplet, sept = septuplet, br = broad signal, m = multiplet), coupling constants (*J*) in Hz and integration. For the assignment of the ^{1}H and ^{13}C{^{1}H} NMR spectra COSY, HSQC and HMBC experiments were also performed. Elemental analysis was carried out by the analytical services of London Metropolitan University. UV–vis spectra were recorded in a Varian CaryWin 300Bio UV-Visible spectrophotometer at concentrations of ca. 0.13 mg/mL of complexes **2–12** in CH_2Cl_2. Fluorescence spectra (λ_{max}/excitation (nm) and λ_{max}/emission (nm)) were recorded in a Cary Eclipse Fluorimeter (settings: 5 nm excitation/emission slit, 120 nm/min scan) at concentrations of ca. 3 mg/mL of complexes **2–12** in CH_2Cl_2. CCDC 949310 (**2**), 949311 (**3**), 949312 (**7**), 949313 (**8**), 949314 (**10**), 949315 (**11**) and 949316 (**12**) contain the supplementary crystallographic data for this paper. These data can be obtained free of charge from The Cambridge Crystallographic Data Centre (CCDC) via http://www.ccdc.cam.ac.uk/data_request/cif.

Synthesis of (1,3-bis(2,6-diisopropylphenyl)imidazol-2-ylidene)(phenylamino)gold Complex (2):

[Au(IPr)(OH)] (100 mg, 166 μmol) and aniline (15.5 mg, 166 μmol) were dissolved in THF (1.5 mL). The reaction mixture was stirred at room temperature for 20 h. The solvent was then concentrated under vacuum (until ca. 0.3 mL) and the product precipitated by addition of pentane (3 mL), filtered, washed with more pentane (4 × 4 mL) and dried under high vacuum. Complex **2** was obtained as a white solid in 66% yield (73 mg).

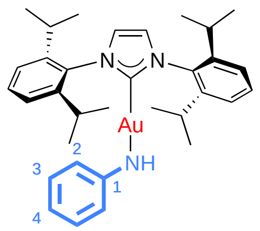

^{1}H NMR (300 MHz; CD_2Cl_2): δ 7.57 (t, *J* = 7.8 Hz, 2H, $CH_{p\text{-Ar}}$), 7.36 (d, *J* = 7.8 Hz, 4H, $CH_{m\text{-Ar}}$), 7.22 (s, 2H, $CH_{imid.}$), 6.61 (dd, *J* = 8.5, 7.1 Hz, 2H,), 6.03 (tt, *J* = 7.1, 1.1 Hz, 1H), 5.95 (dd, *J* = 8.5, 1.1 Hz, 2H), 3.44 (bs, 1H), 2.64 (sept, *J* = 6.9 Hz, 4H), 1.35 (d, *J* = 6.9 Hz, 12H), 1.24 (d, *J* = 6.9 Hz, 12H).

^{13}C{^{1}H} NMR (101 MHz, CD_2Cl_2): δ 175.51 (C_{carb}), 159.43 (C^1), 146.15 ($C_{o\text{-Ar}}$), 134.42 ($C_{N\text{-Ar}}$), 131.07 ($CH_{p\text{-Ar}}$), 130.70 (CH^3), 124.65 ($CH_{m\text{-Ar}}$), 124.48 (CH^4), 123.69 ($CH_{imid.}$), 114.86 (CH^2), 29.19 (CH_{iPr}), 24.56 (CH_3), 24.13 (CH_3).

Anal. Calcd. for $C_{33}H_{42}AuN_3$ (677.67): C, 58.49; H, 6.25; N, 6.20. Found: C, 58.41; H, 6.22; N, 6.28.

Synthesis of (1,3-bis(2,6-diisopropylphenyl)imidazol-2-ylidene)(pyridin-2-ylamino)gold complex (3):

[Au(IPr)(OH)] (30 mg, 50 μmol) and 2-aminopyridine (4.9 mg, 52 μmol) were dissolved in THF (0.5 mL). The reaction mixture was stirred at room temperature for 20 h. The product was then precipitated by addition of pentane (3 mL), filtered, washed with more pentane (4 × 4 mL) and dried under high vacuum. Complex **3** was obtained as a white solid in 94% yield (32 mg).

^{1}H NMR (400 MHz; CD_2Cl_2): δ 7.50 (t, *J* = 7.8 Hz, 2H, $CH_{p\text{-Ar}}$ + m, 1H, CH^5), 7.36 (d, *J* = 7.8 Hz, 4H, $CH_{m\text{-Ar}}$), 7.24 (s, 2H, $CH_{imid.}$), 6.75 (t, *J* = 8.4 Hz, 1H, CH^3), 5.94 (m, 1H, CH^4), 5.59 (d, *J* = 8.5 Hz, 1H,

CH^2), 4.20 (s, 1H, N*H*), 2.62 (sept, *J* = 6.9 Hz, 4H, CH_{iPr}), 1.34 (d, *J* = 6.9 Hz, 12H, CH_3), 1.24 (d, *J* = 6.9 Hz, 12H, CH_3).

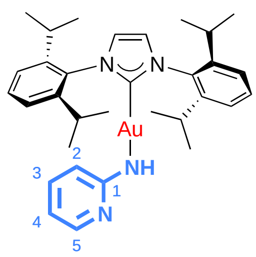

^{13}C{^{1}H} NMR (126 MHz, CD_2Cl_2): δ 179.61 (C_{carb}), 168.96 (C^1), 147.92 (CH^5), 146.41 ($C_{o\text{-}Ar}$), 135.63 (CH^3), 134.81 ($C_{N\text{-}Ar}$), 130.76 ($CH_{p\text{-}Ar}$), 124.50 ($CH_{m\text{-}Ar}$), 123.49 ($CH_{imid.}$), 109.94 (CH^2), 107.58 (CH^4), 29.19 (CH_{iPr}), 24.41 (CH_3), 24.19 (CH_3).

Anal. Calcd. for $C_{32}H_{41}AuN_4$ (678.66): C, 56.63; H, 6.09; N, 8.26. Found: C, 56.55; H, 5i.98; N, 8.11.

Synthesis of (1,3-bis(2,6-diisopropylphenyl)imidazol-2-ylidene)(pyridin-3-ylamino)gold Complex (4):

[Au(IPr)(OH)] (30 mg, 50 μmol) and 3-aminopyridine (4.9 mg, 52 μmol) were dissolved in THF (0.5 mL). The reaction mixture was stirred at room temperature for 20 h. The product was then precipitated by addition of pentane (3 mL), filtered, washed with more pentane (4 × 4 mL) and dried under high vacuum. Complex **4** was obtained as a white solid in 85% yield (29 mg).

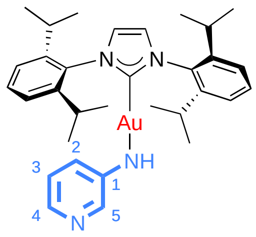

^{1}H NMR (400 MHz; CD_2Cl_2): δ 7.57 (t, *J* = 7.8 Hz, 2H, $CH_{p\text{-}Ar}$), 7.40 (d, *J* = 2.9 Hz, 1H, CH^5), 7.36 (d, *J* = 7.9 Hz, 4H, $CH_{m\text{-}Ar}$), 7.26 (dd, *J* = 4.5, 1.1 Hz, 1H, CH^4), 7.23 (s, 2H, $CH_{imid.}$), 6.51 (dd, *J* = 8.4, 4.5 Hz, 1H, CH^3), 6.12 (dd, *J* = 8.3, 1.6 Hz, 1H, CH^2), 3.41 (s, 1H, N*H*), 2.63 (sept, *J* = 6.8 Hz, 4H, CH_{iPr}), 1.34 (d, *J* = 6.9 Hz, 12H, CH_3), 1.24 (d, *J* = 6.9 Hz, 12H, CH_3).

^{13}C{^{1}H} NMR (126 MHz, CD_2Cl_2): δ 179.85 (C_{carb}), 155.41 (C^1), 146.38 ($C_{o\text{-}Ar}$), 137.95 (CH^3), 134.81 ($C_{N\text{-}Ar}$), 132.73 (CH^4), 130.79 ($CH_{p\text{-}Ar}$), 124.50 ($CH_{m\text{-}Ar}$), 123.47 ($CH_{imid.}$), 123.21 (CH^3), 119.67 (CH^2), 29.20 (CH_{iPr}), 24.42 (CH_3) 24.18 (CH_3).

Anal. Calcd. for $C_{32}H_{41}AuN_4$ (678.66): C, 56.63; H, 6.09; N, 8.26. Found: C, 56.76; H, 5.98; N, 8.16.

Synthesis of (1,3-bis(2,6-diisopropylphenyl)imidazol-2-ylidene)(pyridin-4-ylamino)gold complex (5):

[Au(IPr)(OH)] (30 mg, 50 μmol) and 4-aminopyridine (4.9 mg, 52 μmol) were dissolved in THF (0.5 mL). The reaction mixture was stirred at room temperature for 20 h. The product was then precipitated by addition of pentane (3 mL), filtered, washed with more pentane (4 × 4 mL) and dried under high vacuum. Complex **9a** was obtained as a white solid in 88% yield (30 mg).

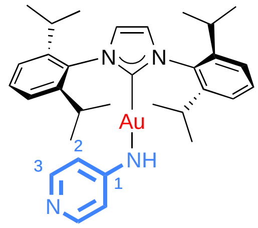

^{1}H NMR (400 MHz; CD_2Cl_2): δ 7.58 (t, *J* = 7.8 Hz, 2H, $CH_{p\text{-}Ar}$), 7.51 (bs, 1H, CH^3), 7.37 (d, *J* = 7.8 Hz, 4H, $CH_{m\text{-}Ar}$), 7.26 (s, 2H, $CH_{imid.}$), 5.76 (bs, 2H, CH^2), 3.89 (bs, 1H, N*H*), 2.61 (sept, *J* = 6.9 Hz, 4H, CH_{iPr}), 1.34 (d, *J* = 6.9 Hz, 12H, CH_3), 1.24 (d, *J* = 6.9 Hz, 12H, CH_3).

^{13}C{^{1}H} NMR (101 MHz, CD_2Cl_2): δ 179.23 (C_{carb}), 164.15 (C^1), 148.84 (CH^3), 146.41 ($C_{o\text{-}Ar}$), 134.73 ($C_{N\text{-}Ar}$), 130.85 ($CH_{p\text{-}Ar}$), 124.54 ($CH_{m\text{-}Ar}$), 123.60 ($CH_{imid.}$), 110.74 (CH^2), 29.21 (CH_{iPr}), 24.43 (CH_3), 24.18 (CH_3).

Anal. Calcd. for $C_{32}H_{41}AuN_4$ (678.66): C, 56.63; H, 6.09; N, 8.26. Found: C, 56.83; H, 5.93; N, 8.34.

Synthesis of (1,3-bis(2,6-diisopropylphenyl)imidazol-2-ylidene)((5-methylpyridin-2-yl)amino)gold Complex (6):

[Au(IPr)(OH)] (30 mg, 50 µmol) and 2-amino-5-methylpyridine (5.6 mg, 52 µmol) were dissolved in THF (0.5 mL). The reaction mixture was stirred at room temperature for 20 h. The product was then precipitated by addition of pentane (3 mL), filtered, washed with more pentane (4 × 4 mL) and dried under high vacuum. Complex **6** was obtained as a white solid in 81% yield (28 mg).

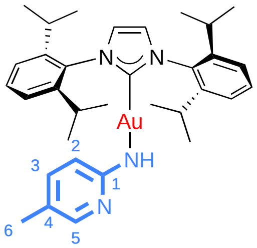

^{1}**H NMR** (400 MHz; CD_2Cl_2): δ 7.57 (t, J = 7.8 Hz, 2H, C$H_{p\text{-Ar}}$), 7.36 (d, J = 7.8 Hz, 4H, C$H_{m\text{-Ar}}$ + 1H, CH^4), 7.24 (s, 2H, C$H_{\text{imid.}}$), 6.61 (dd, J = 8.4, 2.2 Hz, 1H, CH^3), 5.53 (d, J = 8.4 Hz, 1H, CH^2), 4.06 (bs, 1H, NH), 2.63 (sept, J = 6.9 Hz, 4H, C$H_{i\text{Pr}}$), 1.95 (s, 3H, C$H_3{}^6$), 1.34 (d, J = 6.9 Hz, 12H, CH_3), 1.24 (d, J = 6.9 Hz, 12H, CH_3).

^{13}**C{^{1}H} NMR** (101 MHz, CD_2Cl_2): δ 179.96 (C_{carb}), 167.18 (C^1), 147.22 (CH^5), 146.44 (CH$_{o\text{-Ar}}$), 136.95 (CH^3), 134.88 (CH$_{\text{N-Ar}}$), 130.74 (CH$_{p\text{-Ar}}$), 124.51 (CH$_{m\text{-Ar}}$), 123.44 (CH$_{\text{imid.}}$), 115.79 (C^4), 109.58 (CH^2), 29.21 (CH$_{i\text{Pr}}$), 24.40 (CH$_3$), 24.20 (CH$_3$), 17.33 (CH$_3{}^6$).

Anal. Calcd. for $C_{33}H_{43}AuN_4$ (692.69): C, 57.22; H, 6.26; N, 8.09. Found: C, 57.08; H, 6.13; N, 8.00.

Synthesis of (1,3-bis(2,6-diisopropylphenyl)imidazol-2-ylidene)((5-chloropyridin-2-yl)amino)gold complex (7):

[Au(IPr)(OH)] (30 mg, 50 µmol) and 2-amino-5-chloropyridine (6.7 mg, 52 µmol) were dissolved in THF (0.5 mL). The reaction mixture was stirred at room temperature for 20 h. The product was then precipitated by addition of pentane (3 mL), filtered, washed with more pentane (4 × 4 mL) and dried under high vacuum. Complex **7** was obtained as a white solid in 87% yield (31 mg).

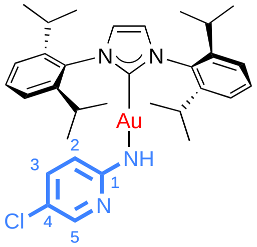

^{1}**H NMR** (400 MHz; CD_2Cl_2): δ 7.57 (t, J = 7.8 Hz, 2H, C$H_{p\text{-Ar}}$), 7.45 (d, J = 2.7, Hz, 1H, CH^5), 7.36 (d, J = 7.8 Hz, 4H, C$H_{m\text{-Ar}}$), 7.24 (s, 2H, C$H_{\text{imid.}}$), 6.68 (dd, J = 9.0, 2.7 Hz, 1H, CH^3), 5.47 (d, J = 8.9 Hz, 1H, CH^2), 4.25 (s, 1H, NH), 2.61 (sept, J = 6.9 Hz, 4H, C$H_{i\text{Pr}}$), 1.33 (d, J = 6.9 Hz, 12H, CH_3), 1.23 (d, J = 6.9 Hz, 12H, CH_3).

^{13}**C{^{1}H} NMR** (126 MHz, CD_2Cl_2): δ 179.06 (C_{carb}), 167.49 (C^1), 146.43 (CH$_{o\text{-Ar}}$), 145.75 (CH^5), 135.34 (CH^3), 134.76 (CH$_{\text{N-Ar}}$), 130.80 (CH$_{p\text{-Ar}}$), 124.53 (CH$_{m\text{-Ar}}$), 123.54 (CH$_{\text{imid.}}$), 113.61 (C^4), 110.96 (CH^2), 29.20 (CH$_{i\text{Pr}}$), 24.41 (CH$_3$), 24.19 (CH$_3$).

Anal. Calcd. for $C_{32}H_{40}AuClN_4$ (713.11): C, 53.90; H, 5.65; N, 7.86. Found: C, 53.84; H, 5.53; N, 7.91.

Synthesis of (1,3-bis(2,6-diisopropylphenyl)imidazol-2-ylidene)((5-(trifluoromethyl)pyridin-2-yl)amino)gold complex (8):

[Au(IPr)(OH)] (30 mg, 50 µmol) and 2-amino-5-(trifluoromethyl)pyridine (7.3 mg, 52 µmol) were dissolved in THF (0.5 mL). The reaction mixture was stirred at room temperature for 20 h. The product was then precipitated by addition of pentane (3 mL), filtered, washed with more pentane (4 × 4 mL) and dried under high vacuum. Complex **8** was obtained as a white solid in 72% yield (27 mg).

^{1}**H NMR** (400 MHz; CD_2Cl_2): δ 7.80 (s, 1H, CH^5), 7.58 (t, J = 7.8 Hz, 2H, C$H_{p\text{-Ar}}$), 7.37 (d, J = 7.8 Hz, 4H, C$H_{m\text{-Ar}}$), 7.27 (s, 2H, C$H_{\text{imid.}}$), 6.90 (d, J = 8.7 Hz, 1H, CH^3), 5.52 (d, J = 8.9 Hz, 1H, CH^2), 4.67 (s, 1H, NH), 2.61 (sept, J = 6.9 Hz, 4H, C$H_{i\text{Pr}}$), 1.34 (d, J = 6.9 Hz, 12H, CH_3), 1.24 (d, J = 6.9 Hz, 12H, CH_3).

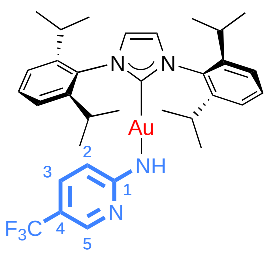

^{13}C{^{1}H} NMR (101 MHz, CD_2Cl_2): δ 178.57 (C_{carb}), 170.62 (C^1), 146.44 ($C_{o\text{-Ar}}$), 146.30 (q, $J_{C\text{-}F}$ = 3.9 Hz, CH^5), 146.15, 134.70 ($C_{N\text{-Ar}}$), 132.28 (q, $J_{C\text{-}F}$ = 2.7 Hz , CH^3), 130.88 ($CH_{p\text{-Ar}}$), 126.49 (q, $J_{C\text{-}F}$ = 258.6 Hz, CF_3), 124.57 ($CH_{m\text{-Ar}}$), 123.66 ($CH_{imid.}$), 109.5 (q, $J_{C\text{-}F}$ = 32.6 Hz, C^4), 109.20 (CH^2), 29.23 (CH_{iPr}), 24.44 (CH_3), 24.20 (CH_3).

^{19}F{^{1}H} NMR (376 MHz, CD_2Cl_2): δ -60.85.

Anal. Calcd. for $C_{33}H_{40}AuF_3N_4$ (746.66): C, 53.08; H, 5.40; N, 7.50. Found: C, 53.02; H, 5.27; N, 7.61.

Synthesis of (1,3-bis(2,6-diisopropylphenyl)imidazol-2-ylidene)((5-nitropyridin-2-yl)amino)gold complex (9):

[Au(IPr)(OH)] (30 mg, 50 µmol) and 2-amino-5-nitropyridine (7.3 mg, 52 µmol) were dissolved in THF (0.5 mL). The reaction mixture was stirred at room temperature for 20 h. The product was then precipitated by addition of pentane (3 mL), filtered, washed with more pentane (4 × 4 mL) and dried under high vacuum. Complex **9** was obtained as a yellow solid in 83% yield (30 mg).

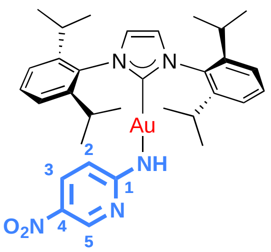

^{1}H NMR (400 MHz; CD_2Cl_2): δ 8.59 (d, J = 2.7 Hz, 1H, CH^5), 7.60 (t, J = 7.8 Hz, 2H, $CH_{p\text{-Ar}}$), 7.51 (dd, J = 9.5, 2.8 Hz, 1H, CH^3), 7.38 (d, J = 7.8 Hz, 4H, $CH_{m\text{-Ar}}$), 7.29 (s, 2H, $CH_{imid.}$), 5.39 (bs, 1H, NH + d, J = 9.5 Hz, 1 H, CH^2), 2.59 (sept, J = 6.9 Hz, 4H, CH_{iPr}), 1.32 (d, J = 6.9 Hz, 12H, CH_3), 1.25 (d, J = 6.9 Hz, 12H, CH_3).

^{13}C{^{1}H} NMR (126 MHz, CD_2Cl_2): δ 177.05 (C_{carb}), 171.23 (C^1), 148.89 (CH^5), 146.41 ($C_{o\text{-Ar}}$), 134.48 ($C_{N\text{-Ar}}$), 132.07 (C^4), 131.30 (CH^3), 131.03 ($CH_{p\text{-Ar}}$), 124.64 ($CH_{m\text{-Ar}}$), 123.87 ($CH_{imid.}$), 109.32 (CH^2), 29.23 (CH_{iPr}), 24.48 (CH_3), 24.18 (CH_3).

Anal. Calcd. for $C_{32}H_{40}AuN_5O_2$ (723.66): C, 53.11; H, 5.57; N, 9.68. Found: C, 53.24; H, 5.50; N, 9.56.

Synthesis of (1,3-bis(2,6-diisopropylphenyl)imidazol-2-ylidene)(isoquinolin-3-ylamino)gold complex (10):

[Au(IPr)(OH)] (30 mg, 50 µmol) and 3-aminoisoquinoline (7.5 mg, 52 µmol) were dissolved in THF (0.5 mL). The reaction mixture was stirred at room temperature for 20 h. The product was then precipitated by addition of pentane (3 mL), filtered, washed with more pentane (4 × 4 mL) and dried under high vacuum. Complex **10** was obtained as a yellow solid in 82% yield (30 mg).

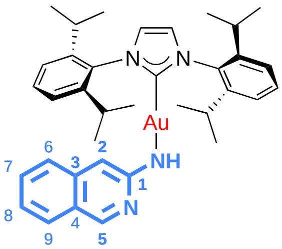

^{1}H NMR (400 MHz; CD_2Cl_2): δ 8.34 (s, 1H, CH^5), 7.62 (t, J = 7.8 Hz, 2H, $CH_{p\text{-Ar}}$), 7.41-7.37 (d, J = 7.8 Hz, 4H, $CH_{m\text{-Ar}}$ + m, 1H, $CH_{isoquinoline}$), 7.25 (s, 2H, $CH_{imid.}$), 7.17 (ddd, J = 8.2, 6.7, 1.3 Hz, 1H, $CH_{isoquinoline}$), 6.95 (d, J = 8.5 Hz, 1H, $CH_{isoquinoline}$), 6.75 (ddd, J = 8.01, 6.71, 1.12 Hz, 1H, $CH_{isoquinoline}$), 5.80 (m, 1H, CH^2), 4.32 (s, 1H, NH), 2.66 (7, J = 6.9 Hz, 4H, CH_{iPr}), 1.37 (d, J = 6.9 Hz, 12H, CH_3), 1.25 (d, J = 6.9 Hz, 12H, CH_3).

^{13}C{^{1}H} NMR (126 MHz, CD_2Cl_2): δ 179.76 (C_{carb}), 166.09 (C^1), 151.24 (CH^5), 146.45 ($C_{o\text{-Ar}}$), 140.01 (C^3), 134.90 ($C_{N\text{-Ar}}$), 130.88 ($CH_{p\text{-Ar}}$), 129.21 ($CH_{isoquinoline}$), 128.07 ($CH_{isoquinoline}$), 124.56 ($CH_{m\text{-Ar}}$), 123.75 ($CH_{isoquinoline}$), 123.52 ($CH_{imid.}$), 121.37 (C^4), 118.73 ($CH_{isoquinoline}$), 96.48 (CH^2), 29.24 (CH_{iPr}), 24.49 (CH_3), 24.18 (CH_3).

Anal. Calcd. for $C_{36}H_{43}AuN_4$ (728.72): C, 59.33; H, 5.95; N, 7.69. Found: C, 59.44; H, 6.02; N, 7.62.

Synthesis of (1,3-bis(2,6-diisopropylphenyl)imidazol-2-ylidene)((5-chloropyrimidin-2-yl)amino)gold complex (11):

[Au(IPr)(OH)] (30 mg, 50 µmol) and 5-chloropyrimidin-2-amine (6.7 mg, 52 µmol) were dissolved in THF (0.5 mL). The reaction mixture was stirred at room temperature for 20 h. The product was then precipitated by addition of pentane (3 mL), filtered, washed with more pentane (4 × 4 mL) and dried under high vacuum. Complex **11** was obtained as a white solid in 87% yield (30 mg).

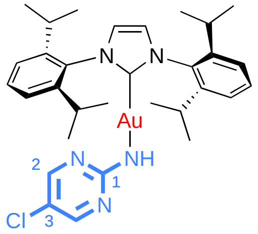

^{1}H NMR (300 MHz; CD_2Cl_2): δ 7.69-7.63 (m, 2H, CH^2), 7.54 (t, J = 7.8 Hz, 2H, C$H_{p\text{-Ar}}$), 7.34 (d, J = 7.8 Hz, 4H, C$H_{m\text{-Ar}}$), 7.22 (s, 2H, C$H_{\text{imid.}}$), 4.54 (s, 1H), 2.68-2.54 (sept, J = 6.9 Hz, 4H, C$H_{i\text{Pr}}$), 1.36 (d, J = 6.9 Hz, 12H, CH_3), 1.23 (d, J = 6.9 Hz, 12H, CH_3).

^{13}C{^{1}H} NMR (75 MHz, CD_2Cl_2): δ 178.55 (C_{carb}), 170.03 (C^1), 155.60 (CH^2), 146.33 ($C_{o\text{-Ar}}$), 134.85 ($C_{N\text{-Ar}}$), 130.73 (CH$_{p\text{-Ar}}$), 124.46 (CH$_{m\text{-Ar}}$), 123.57 (CH$_{\text{imid.}}$), 113.68 (C^3), 29.23 (CH$_{i\text{Pr}}$), 24.33 (CH$_3$), 24.20 (CH$_3$).

Anal. Calcd. for $C_{31}H_{39}AuClN_5$ (714.09): C, 52.14; H, 5.50; N, 9.81. Found: C, 52.26; H, 5.61; N, 9.73.

Synthesis of (1,3-bis(2,6-diisopropylphenyl)imidazol-2-ylidene)(diphenylamino)gold complex (12):

[Au(IPr)(OH)] (30 mg, 50 µmol) and diphenylamine (8.8 mg, 52 µmol) were dissolved in THF (0.5 mL). The reaction mixture was stirred at room temperature for 20 h. The product was then precipitated by addition of pentane (3 mL), filtered, washed with more pentane (4 × 4 mL) and dried under high vacuum. Complex **12** was obtained as a yellow solid in 74% yield (28 mg).

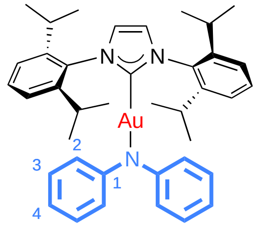

^{1}H NMR (400 MHz; CD_2Cl_2): δ 7.60 (t, J = 7.8 Hz, 2H, C$H_{p\text{-Ar}}$), 7.37 (d, J = 7.8 Hz, 4H, C$H_{m\text{-Ar}}$), 7.27 (s, 2H, C$H_{\text{imid.}}$), 6.79-6.74 (m, 4H, CH^3), 6.65-6.62 (m, 4H, CH^2), 6.40 (tt, J = 7.1, 1.1 Hz, 2H, CH^4), 2.62 (sept, J = 6.9 Hz, 4H, C$H_{i\text{Pr}}$), 1.26 (d, J = 6.9 Hz, 12H, CH_3), 1.23 (d, J = 6.9 Hz, 12H, CH_3).

^{13}C{^{1}H} NMR (101 MHz, CD_2Cl_2): δ 178.32 (C_{carb}), 154.68 (C^1), 146.41 ($C_{o\text{-Ar}}$), 134.94 ($C_{\text{N-Ar}}$), 130.72 (CH$_{p\text{-Ar}}$), 128.45 (CH^3), 124.46 (CH$_{m\text{-Ar}}$), 123.34 (CH$_{\text{imid.}}$), 119.72 (CH^2), 116.59 (CH^4), 29.22 (CH$_{i\text{Pr}}$), 24.35 (CH$_3$), 24.22 (CH$_3$).

Anal. Calcd. for $C_{39}H_{46}AuN_3$ (753.77): C, 62.14; H, 6.15; N, 5.57. Found: C, 62.22; H, 6.29; N, 5.68.

UV–vis and fluorescence spectra:

- Equipment Used:
  - **UV**: Varian CaryWin 300Bio UV–Vis spectrophotometer
  - **Fluorescence**: Cary Eclipse Fluorimeter (settings: 5 nm excitation/emission slit, 120 nm/min scan).

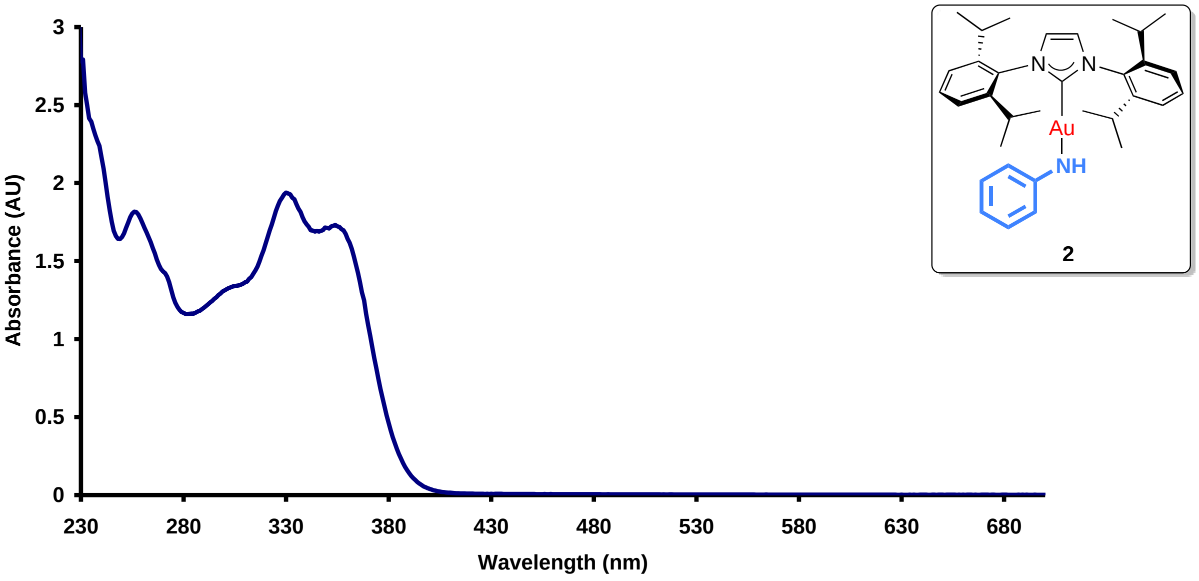

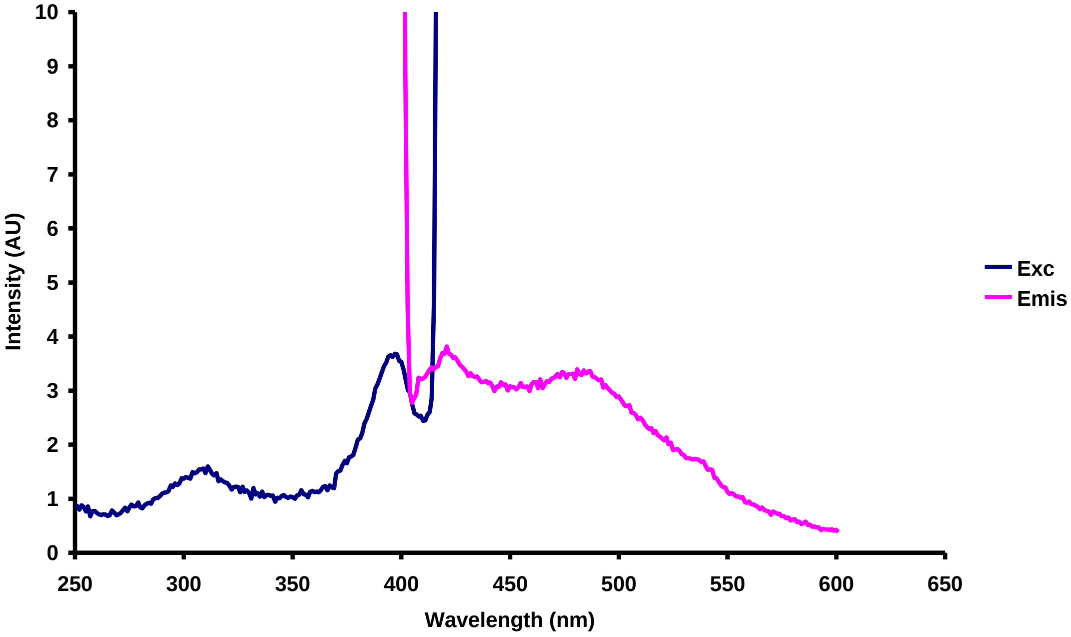

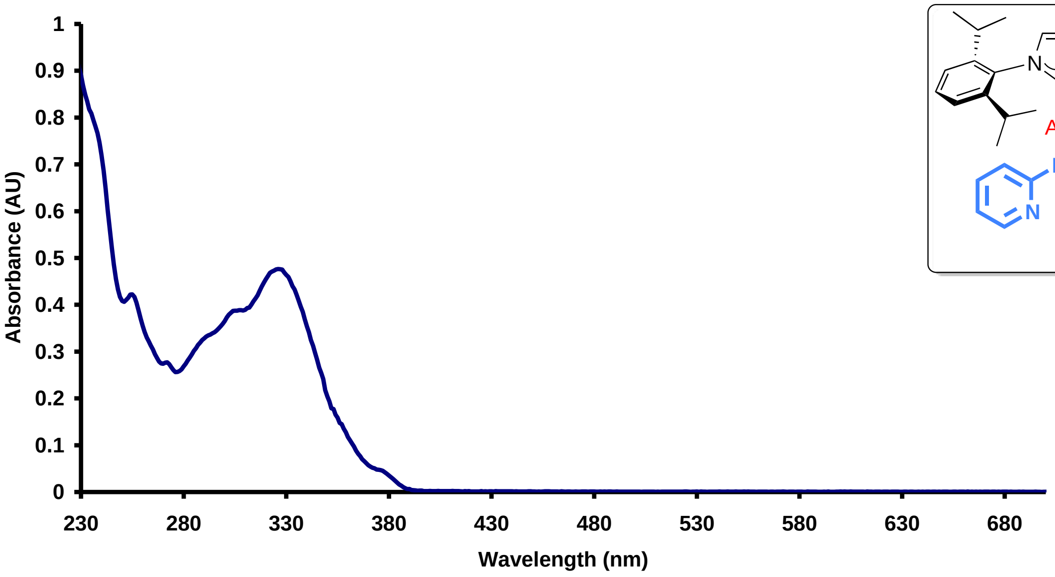
Absorbance (AU)
0
0.1
0.2
0.3
0.4
0.5
0.6
0.7
0.8
0.9
1
230
280
330
380
430
480
530
580
630
680
Wavelength (nm)

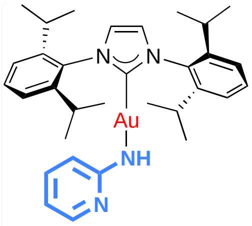
N
N
Au
NH
N

3

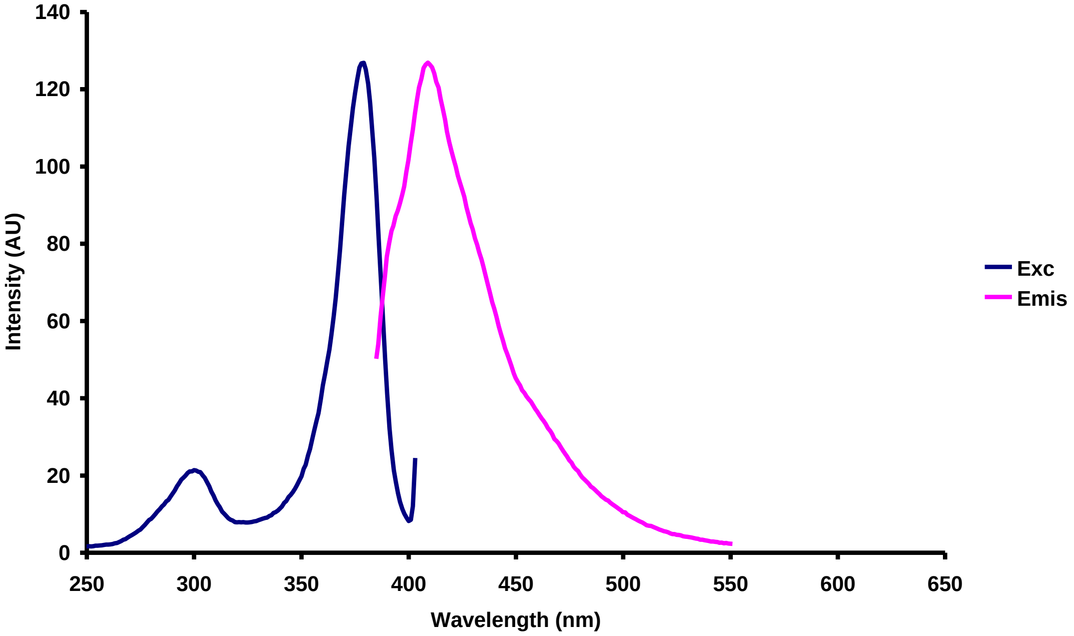
Intensity (AU)
0
20
40
60
80
100
120
140
250
300
350
400
450
500
550
600
650
Wavelength (nm)
Exc
Emis

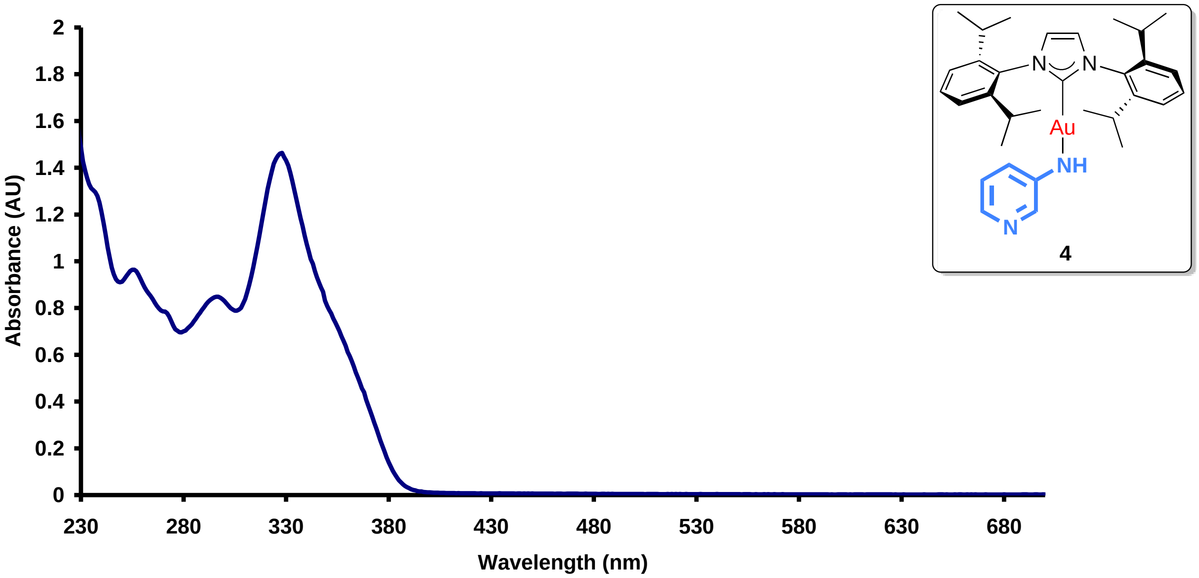
Absorbance (AU)
2
1.8
1.6
1.4
1.2
1
0.8
0.6
0.4
0.2
0
230
280
330
380
430
480
530
580
630
680
Wavelength (nm)
N
N
Au
NH
N
4

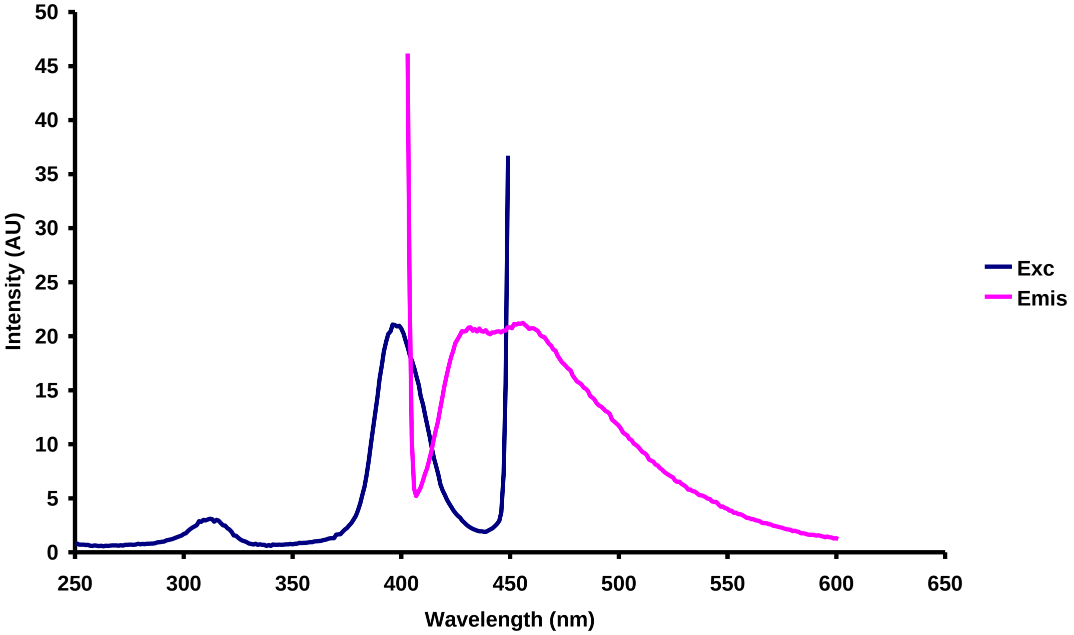
Intensity (AU)
50
45
40
35
30
25
20
15
10
5
0
250
300
350
400
450
500
550
600
650
Wavelength (nm)
Exc
Emis

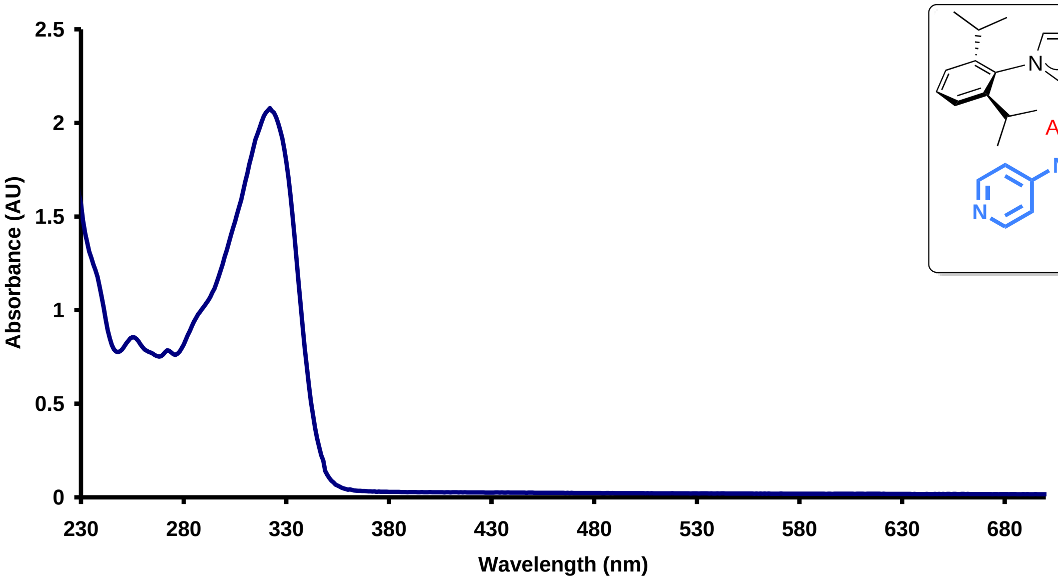
Absorbance (AU)
2.5
2
1.5
1
0.5
0
230
280
330
380
430
480
530
580
630
680
Wavelength (nm)

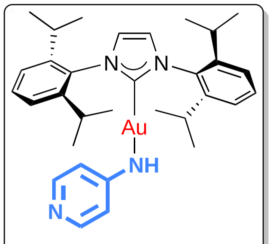
N
N
Au
NH
N

5

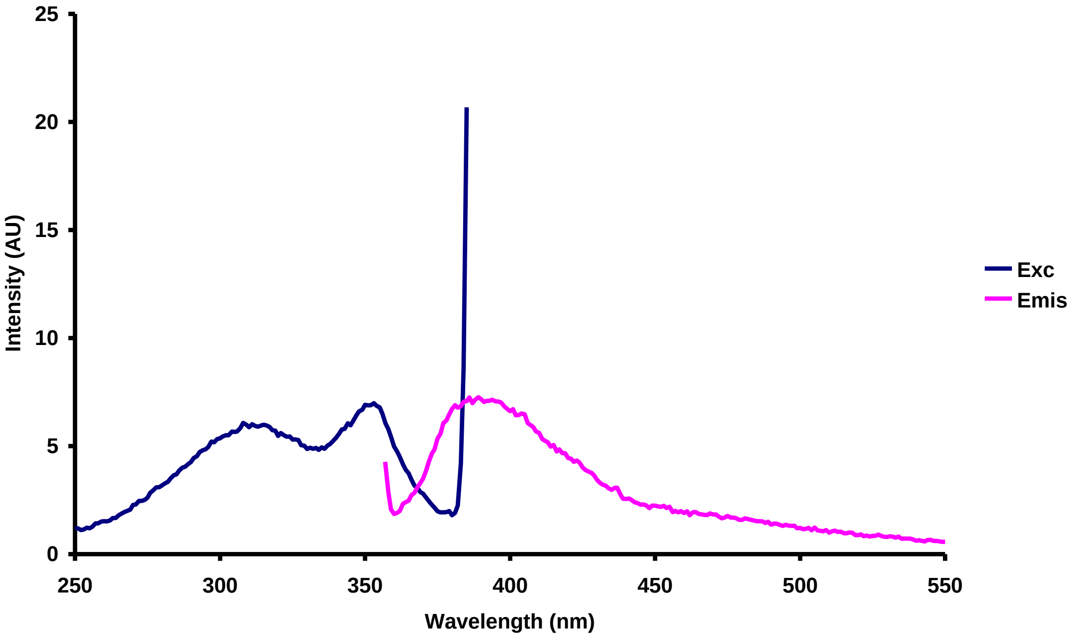
Intensity (AU)
25
20
15
10
5
0
250
300
350
400
450
500
550
Wavelength (nm)
Exc
Emis

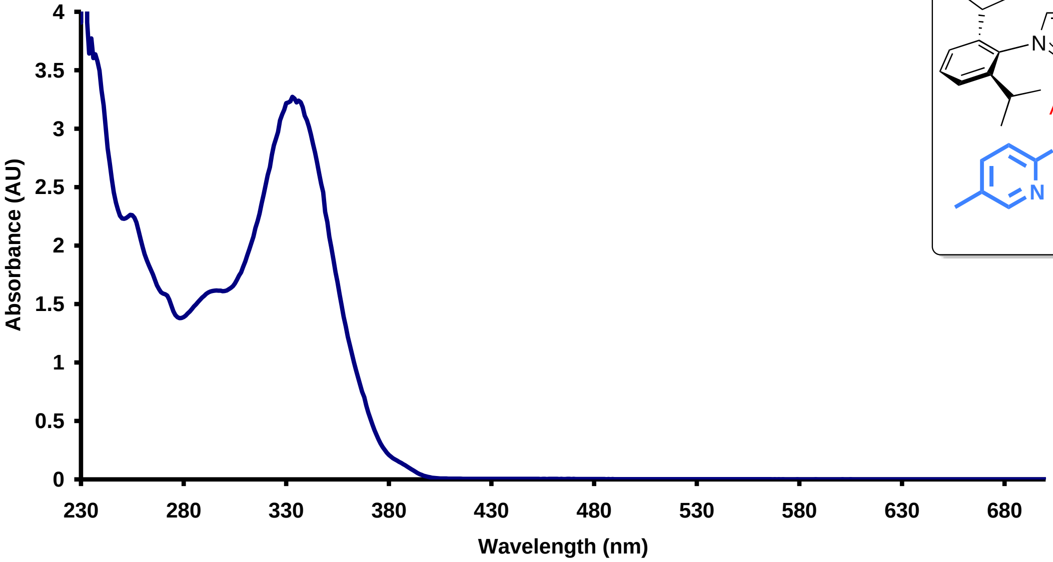
Absorbance (AU)
Wavelength (nm)
0
0.5
1
1.5
2
2.5
3
3.5
4
230
280
330
380
430
480
530
580
630
680

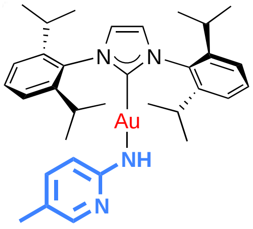
N
N
Au
NH
N

6

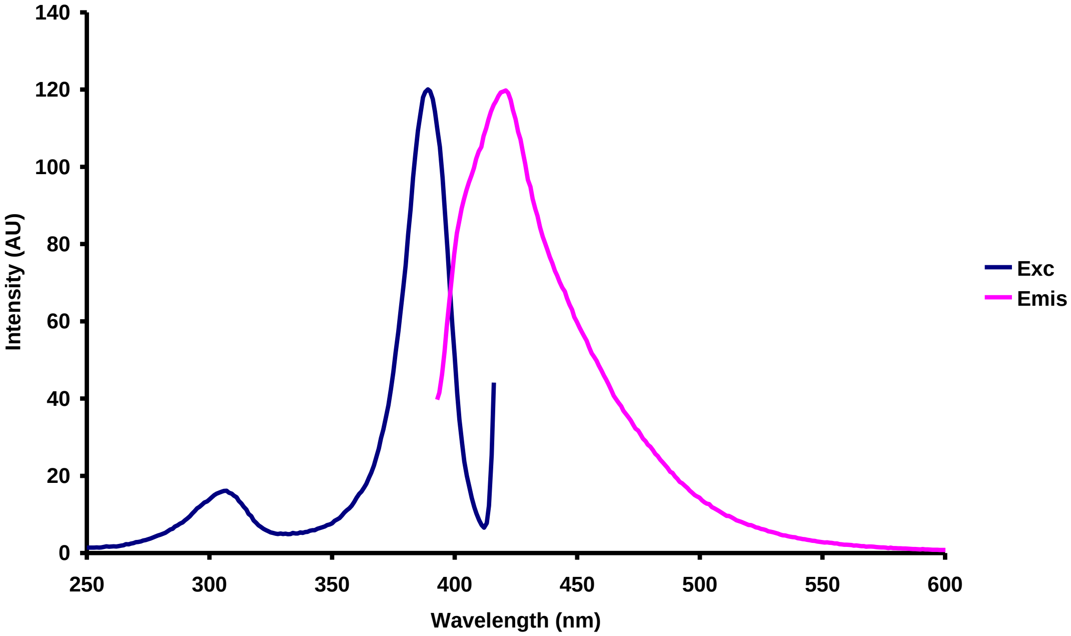
Intensity (AU)
Wavelength (nm)
0
20
40
60
80
100
120
140
250
300
350
400
450
500
550
600
Exc
Emis

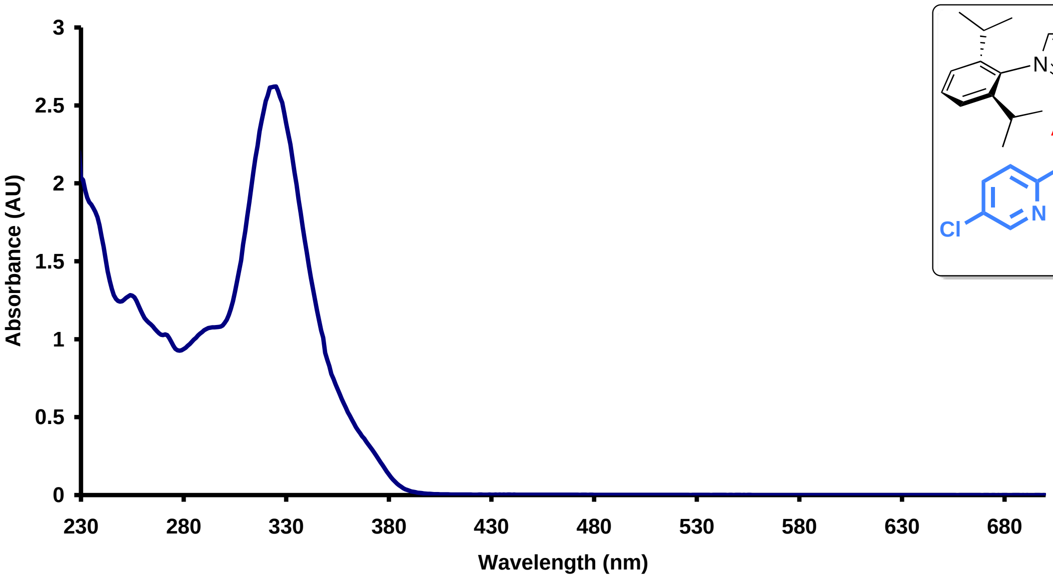
3
2.5
2
1.5
1
0.5
0
Absorbance (AU)
230
280
330
380
430
480
530
580
630
680
Wavelength (nm)

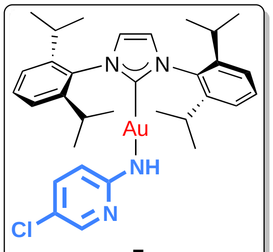
N
N
Au
NH
N
Cl

7

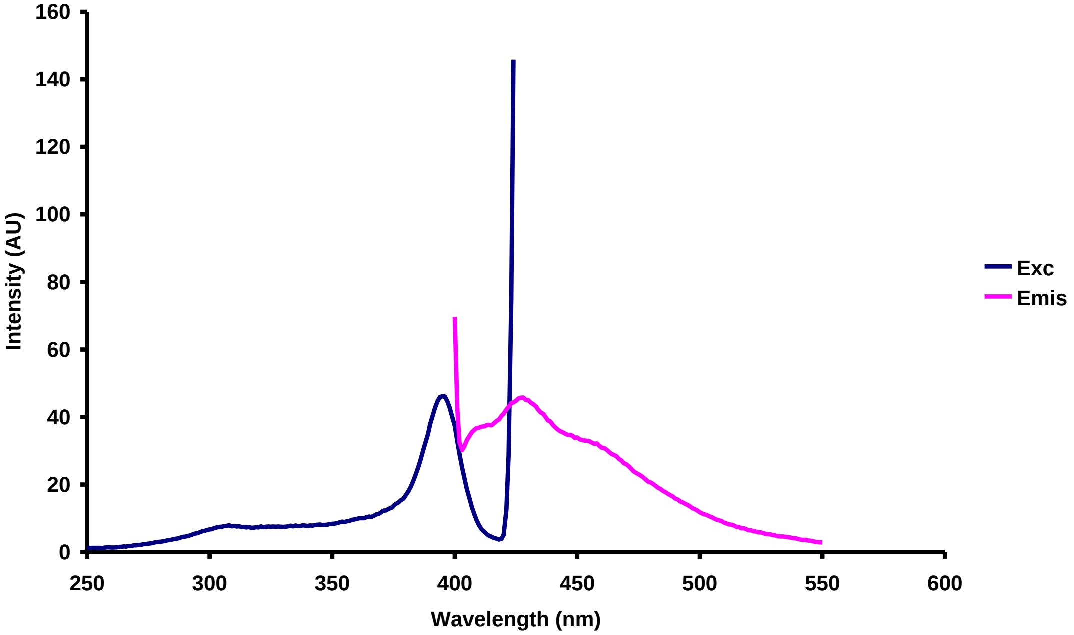
160
140
120
100
80
60
40
20
0
Intensity (AU)
250
300
350
400
450
500
550
600
Wavelength (nm)
Exc
Emis

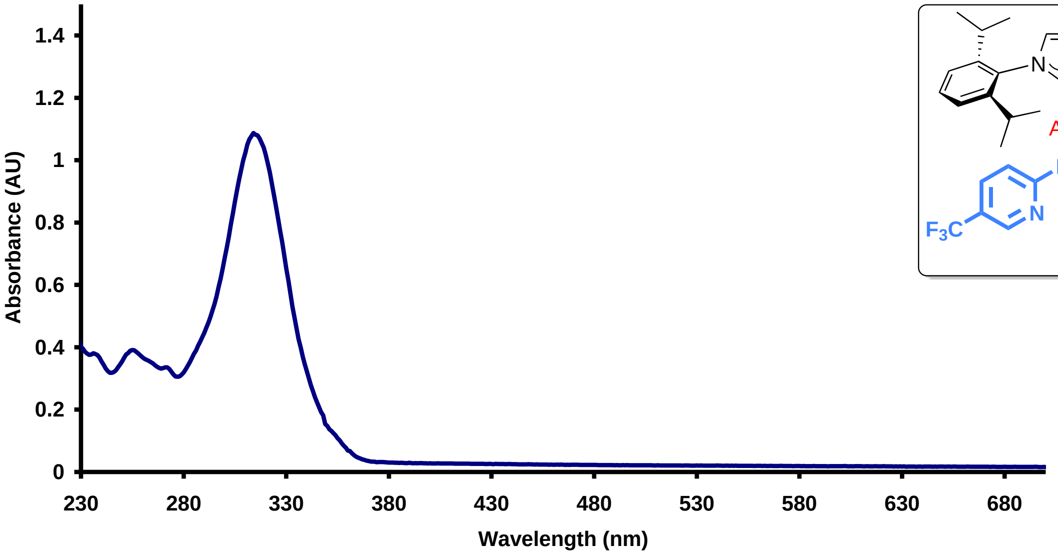

Absorbance (AU)
Wavelength (nm)

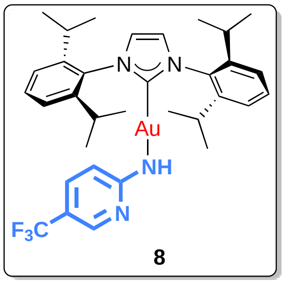

N
N
Au
NH
N
F3C
8

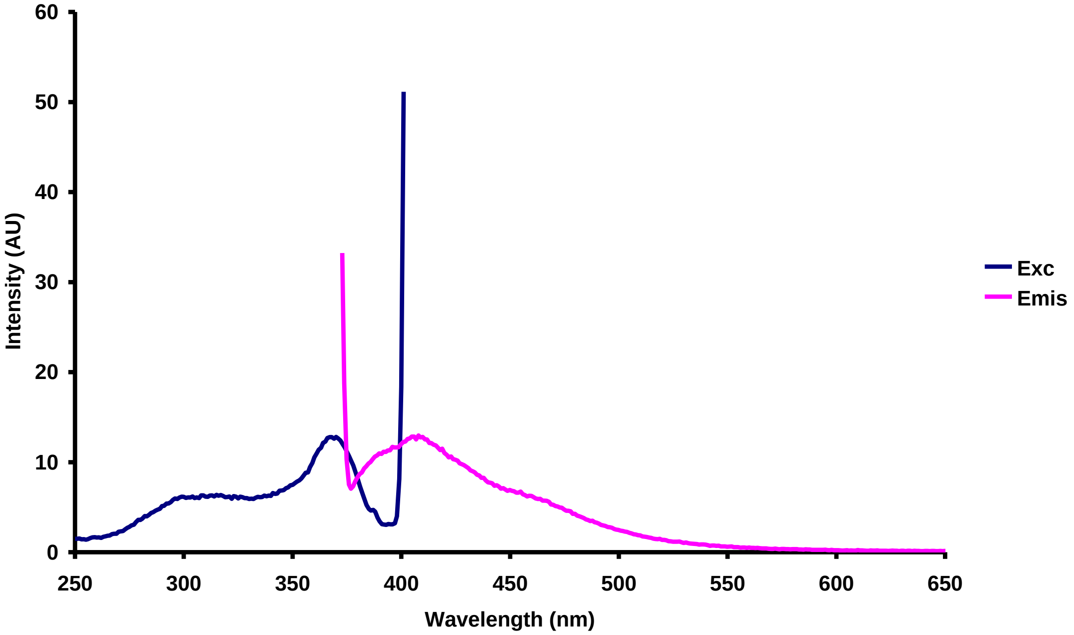

Intensity (AU)
Wavelength (nm)
Exc
Emis

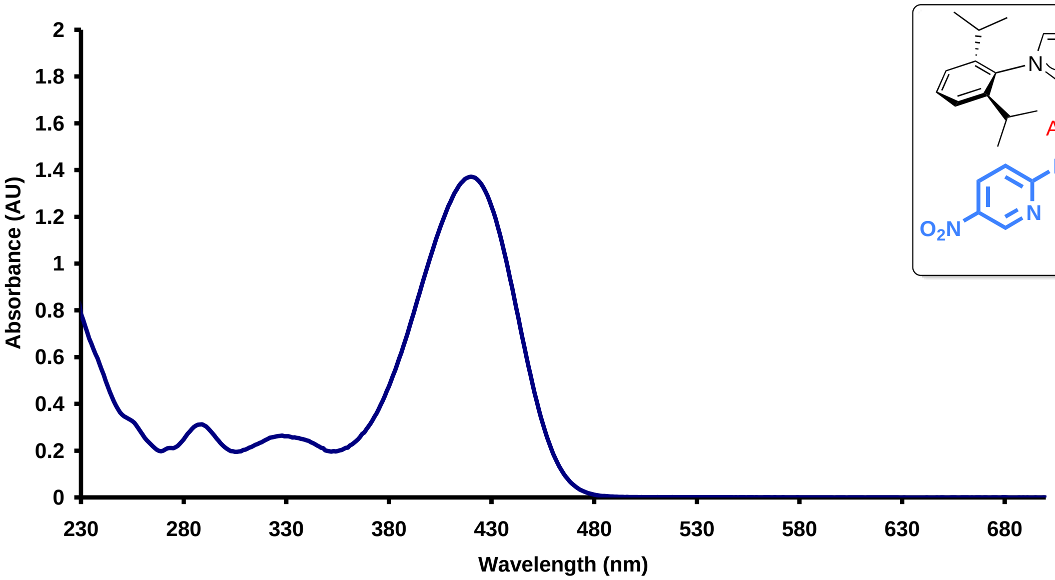
Absorbance (AU)
2
1.8
1.6
1.4
1.2
1
0.8
0.6
0.4
0.2
0
230
280
330
380
430
480
530
580
630
680
Wavelength (nm)

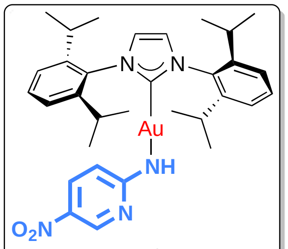
N
N
Au
NH
N
O2N

9

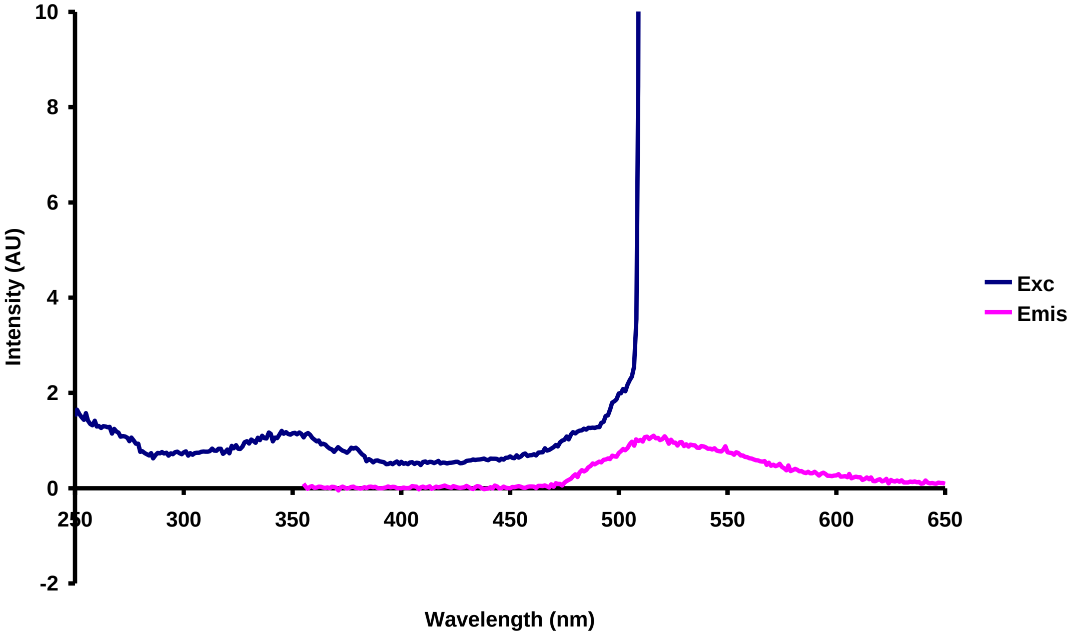
Intensity (AU)
10
8
6
4
2
0
-2
250
300
350
400
450
500
550
600
650
Wavelength (nm)
Exc
Emis

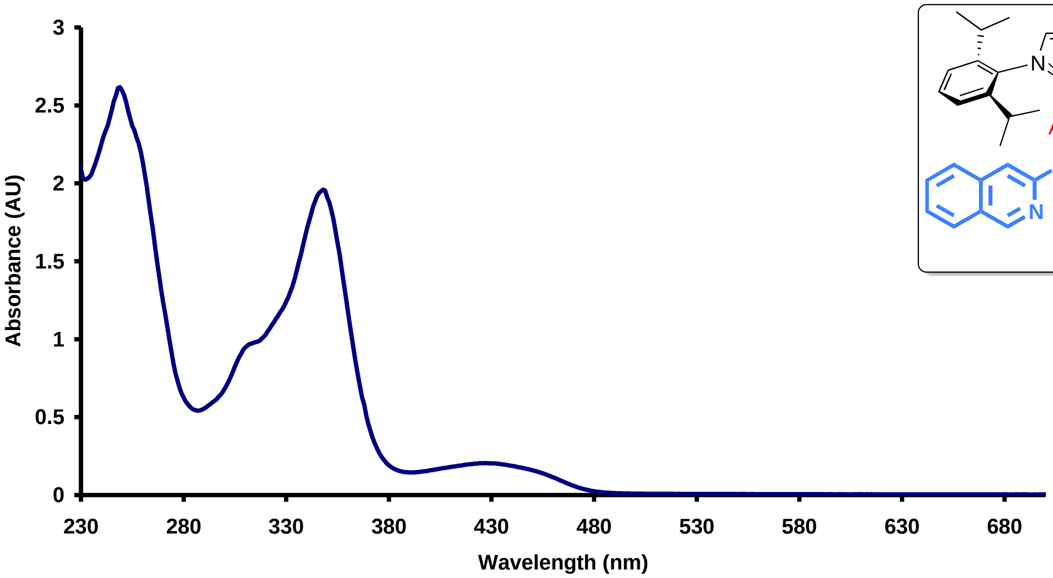
Absorbance (AU)
0
0.5
1
1.5
2
2.5
3
230
280
330
380
430
480
530
580
630
680
Wavelength (nm)

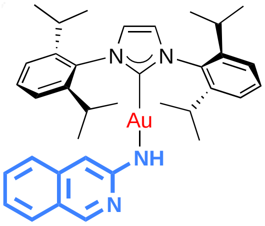
N
N
Au
NH
N

10

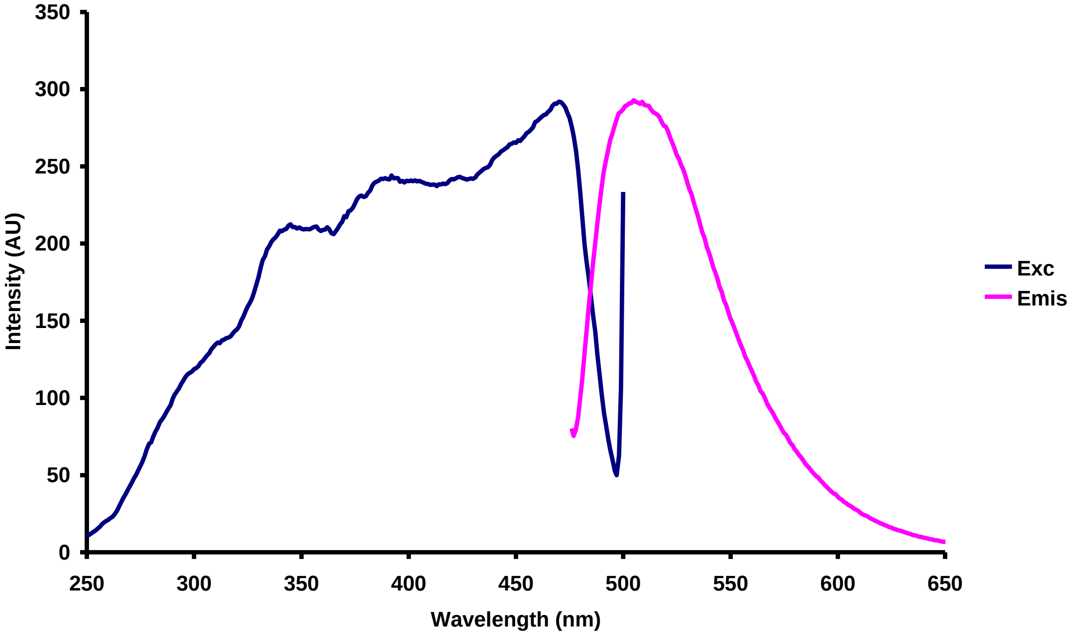
Intensity (AU)
0
50
100
150
200
250
300
350
250
300
350
400
450
500
550
600
650
Wavelength (nm)
Exc
Emis

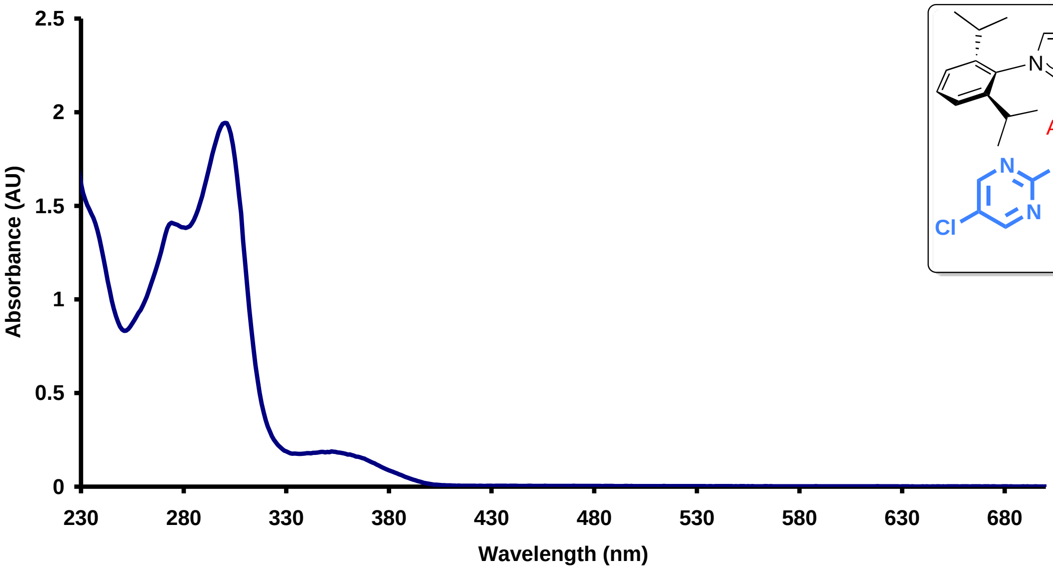
2.5
2
1.5
1
0.5
0
Absorbance (AU)
230
280
330
380
430
480
530
580
630
680
Wavelength (nm)

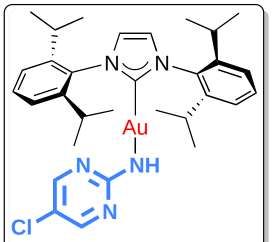
N
N
Au
N
NH
N
Cl

11

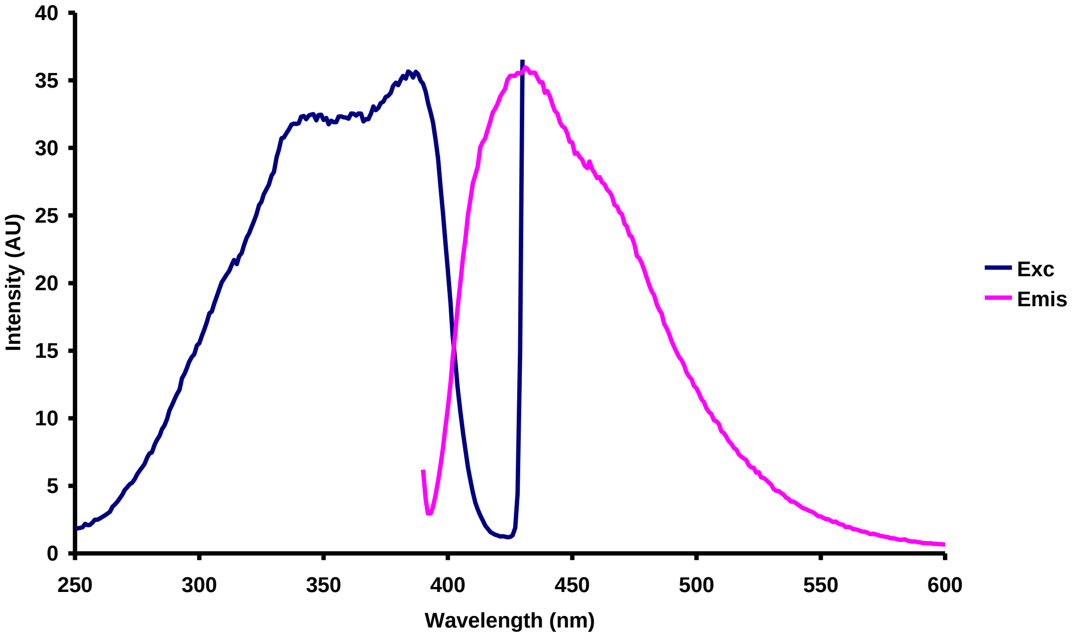
40
35
30
25
20
15
10
5
0
Intensity (AU)
250
300
350
400
450
500
550
600
Wavelength (nm)
Exc
Emis

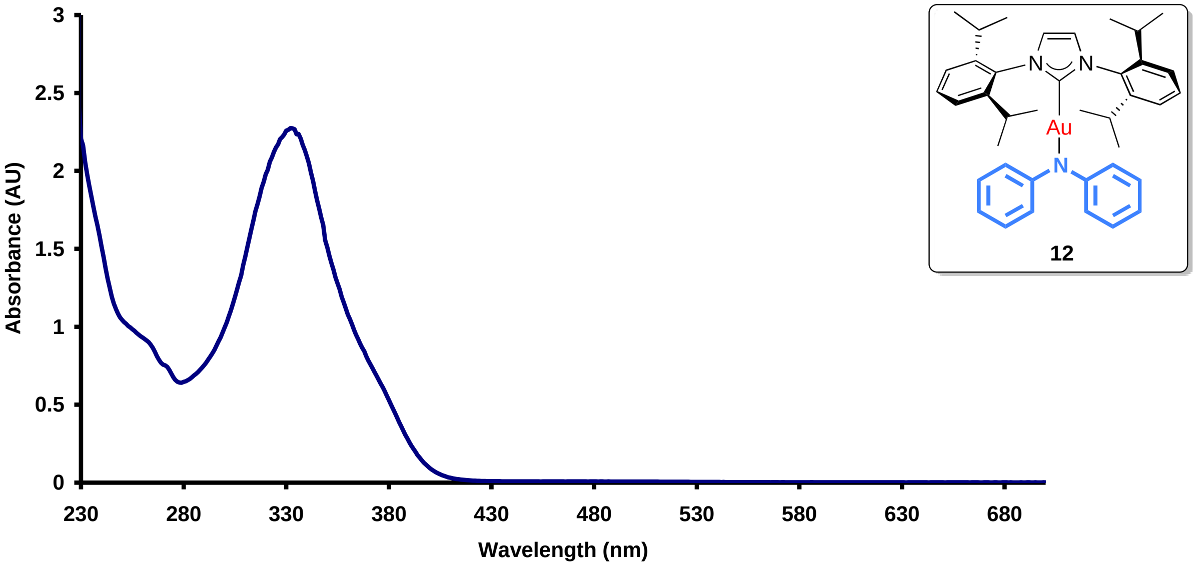
Absorbance (AU)
Wavelength (nm)
12
Au
N

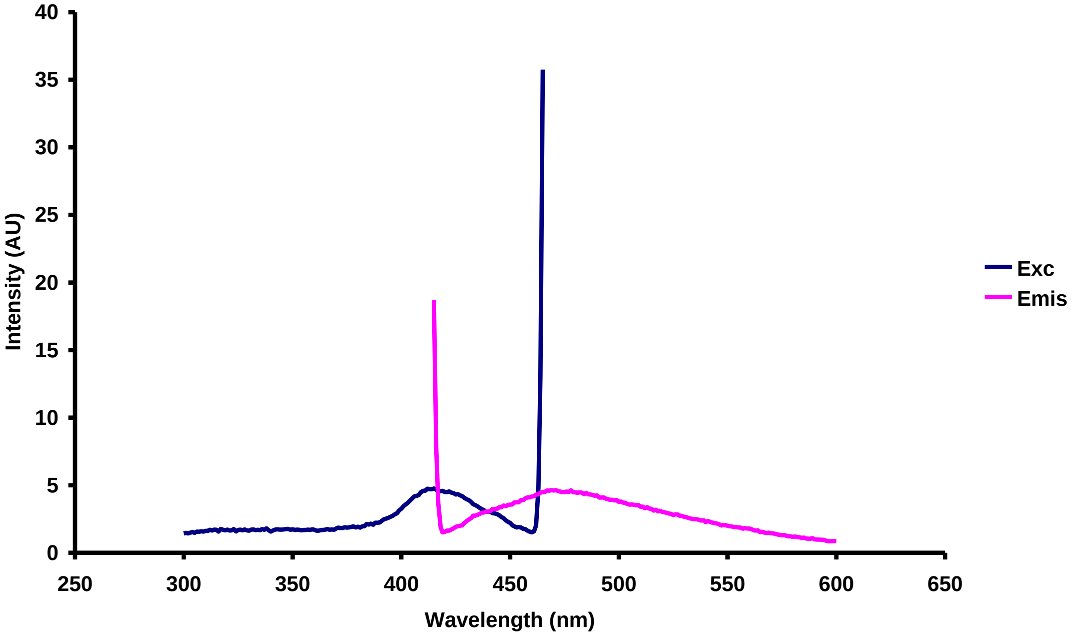
Intensity (AU)
Wavelength (nm)
Exc
Emis

NMR Spectra

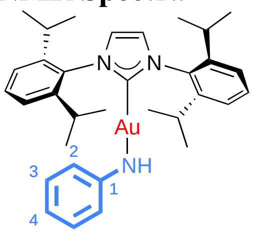

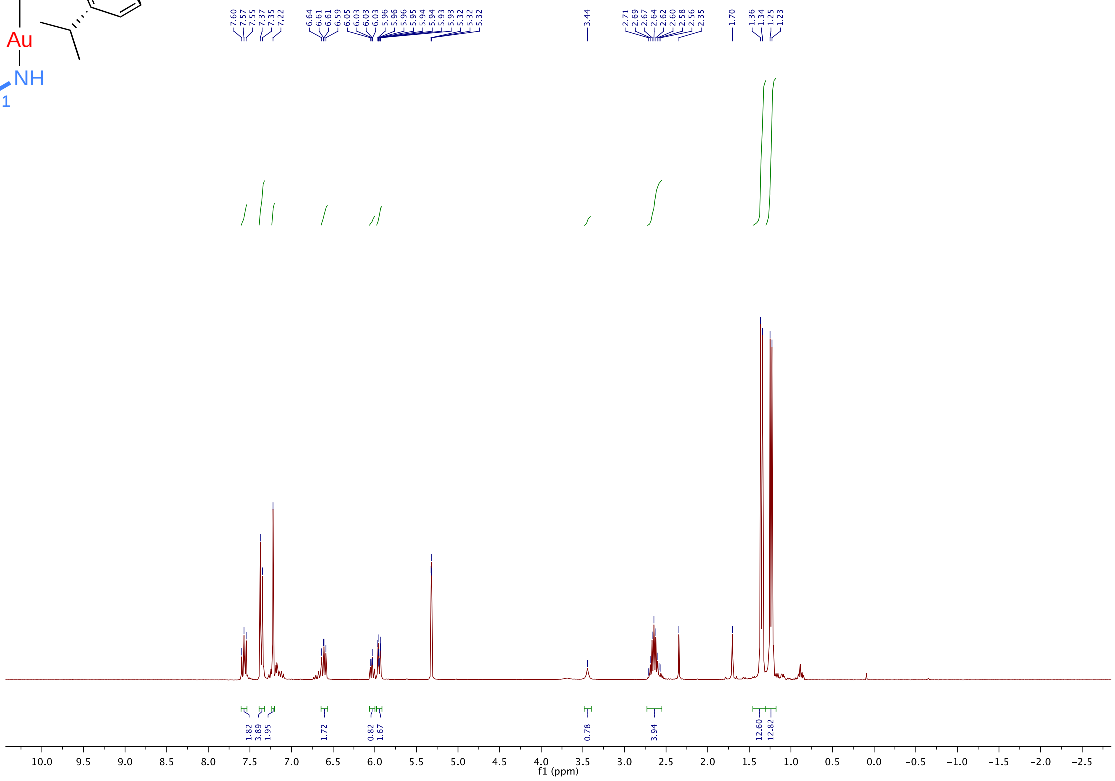

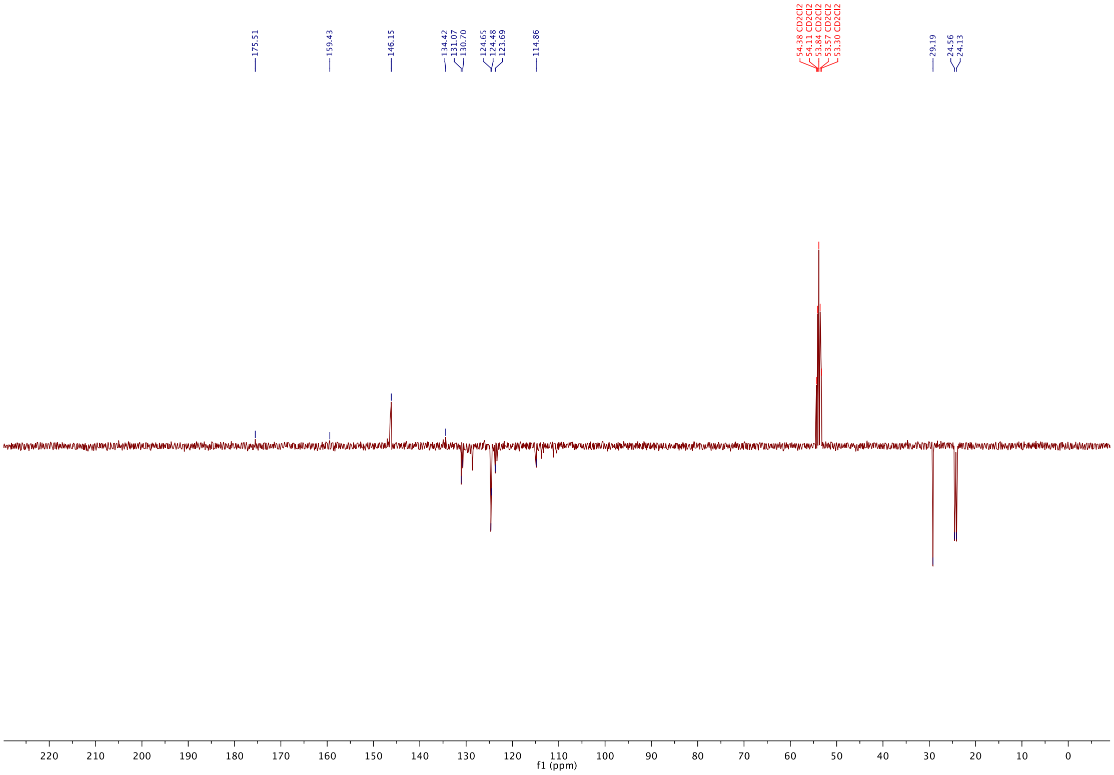

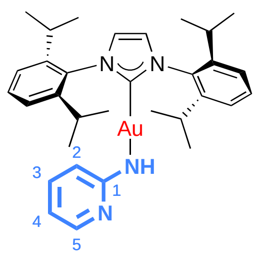

Au
NH
N
N
1
2
3
4
5

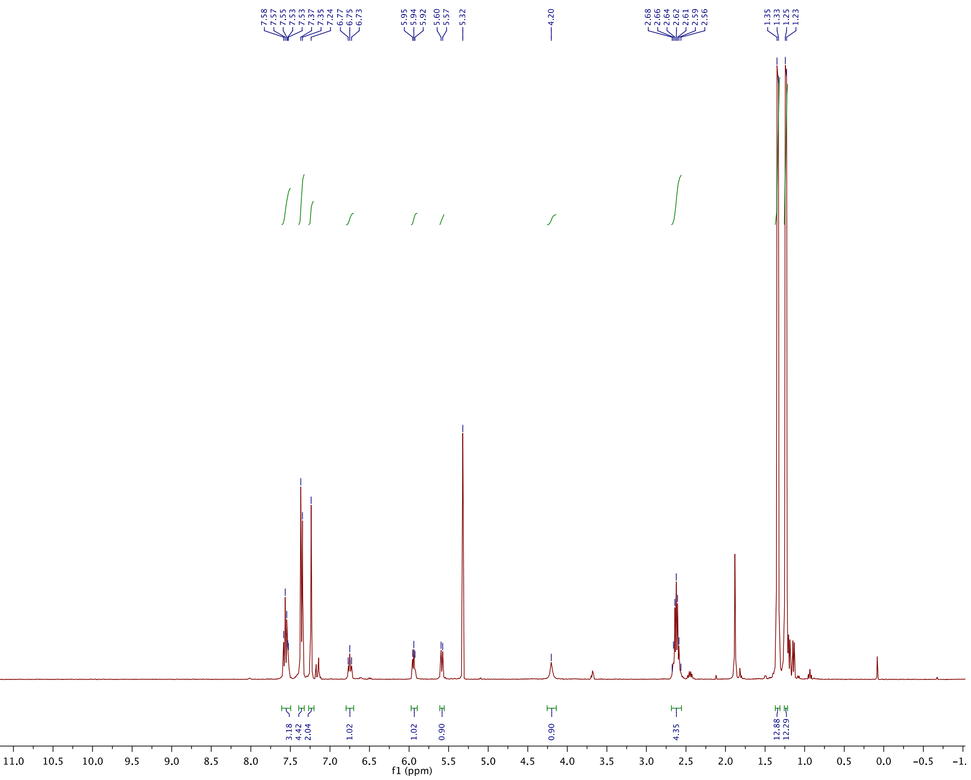

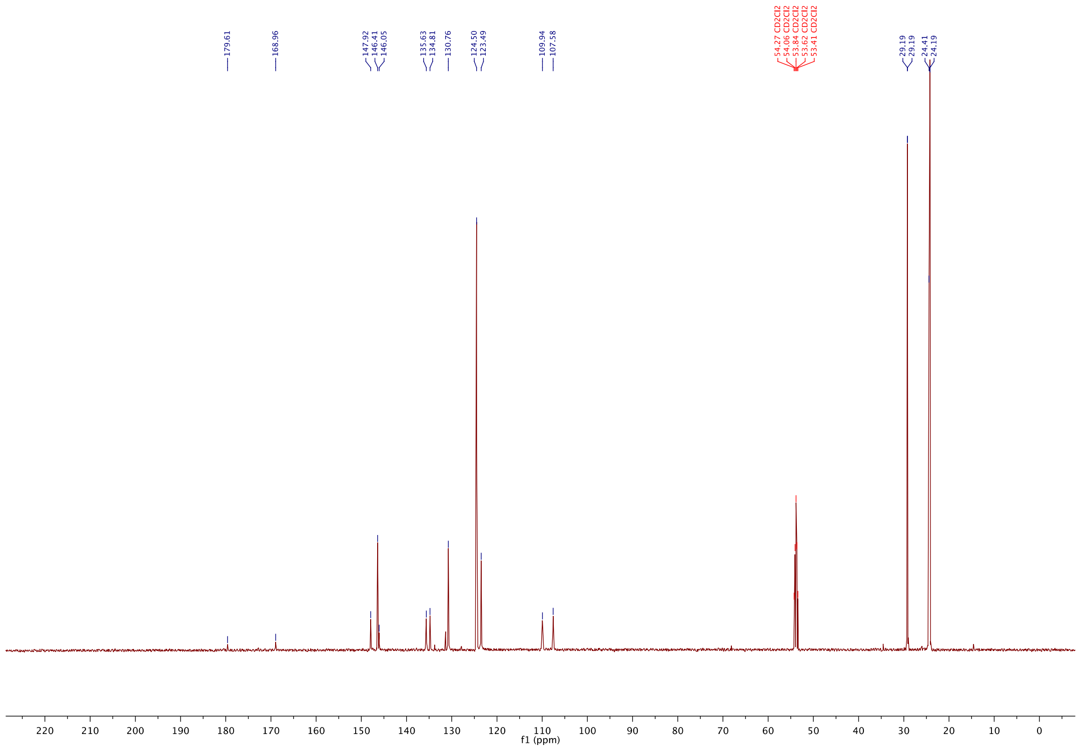

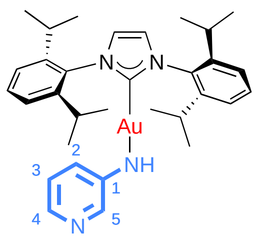

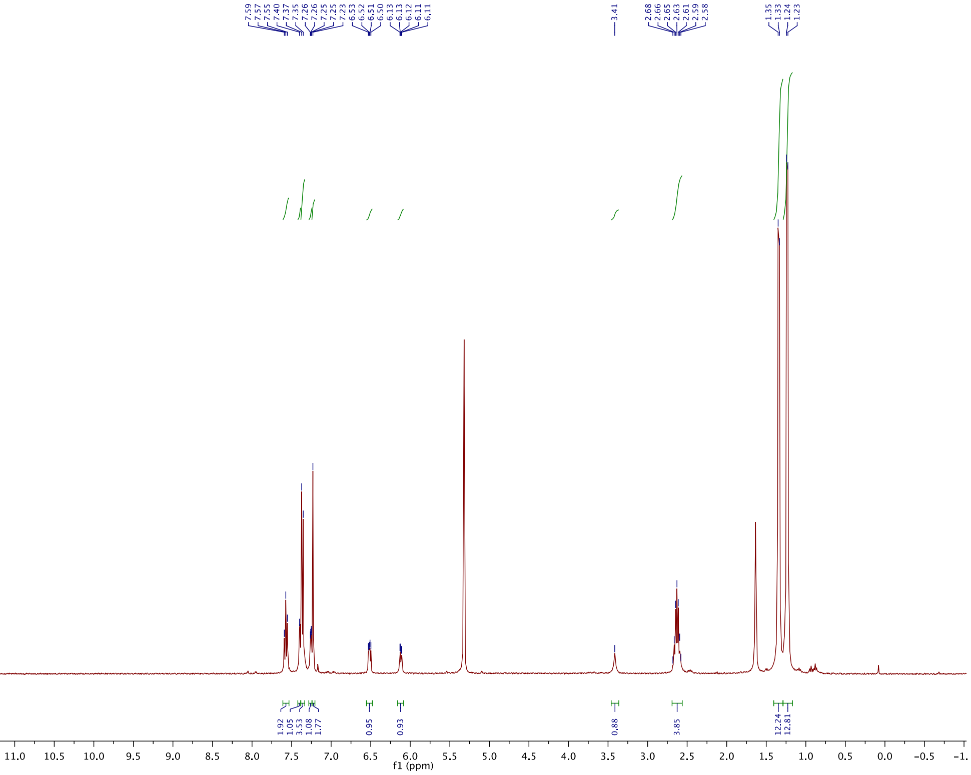

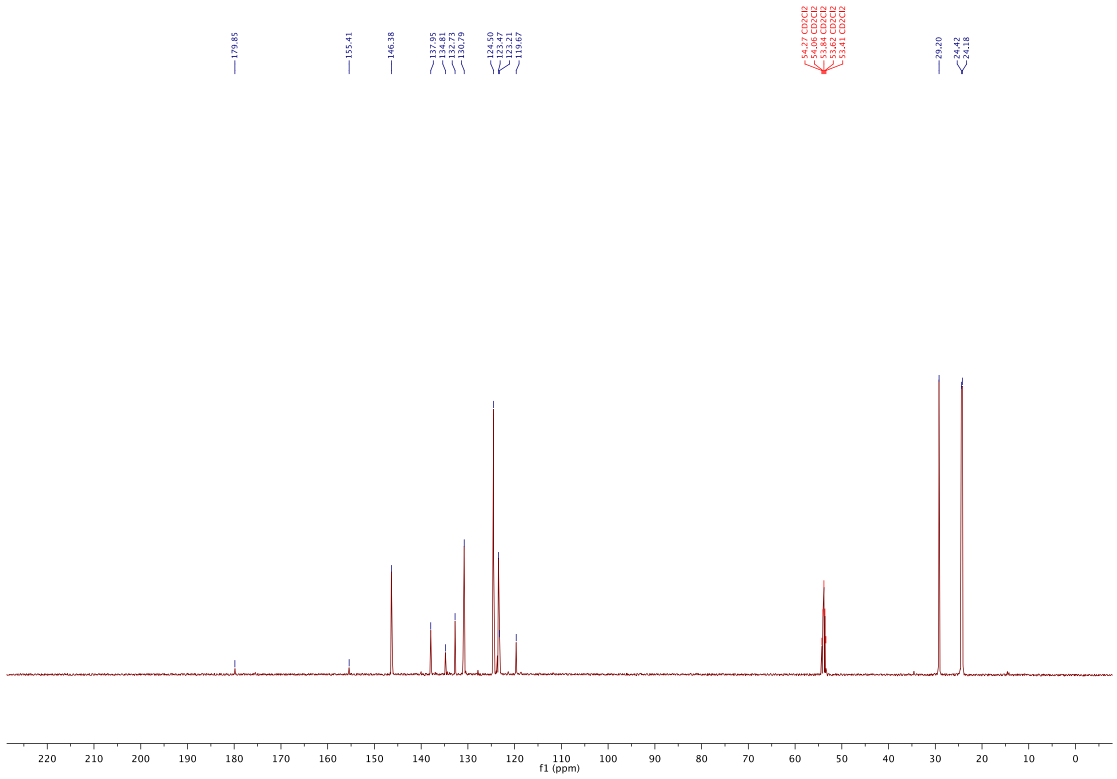

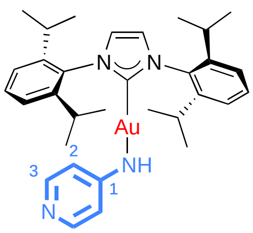
N
N
Au
NH
N
1
2
3

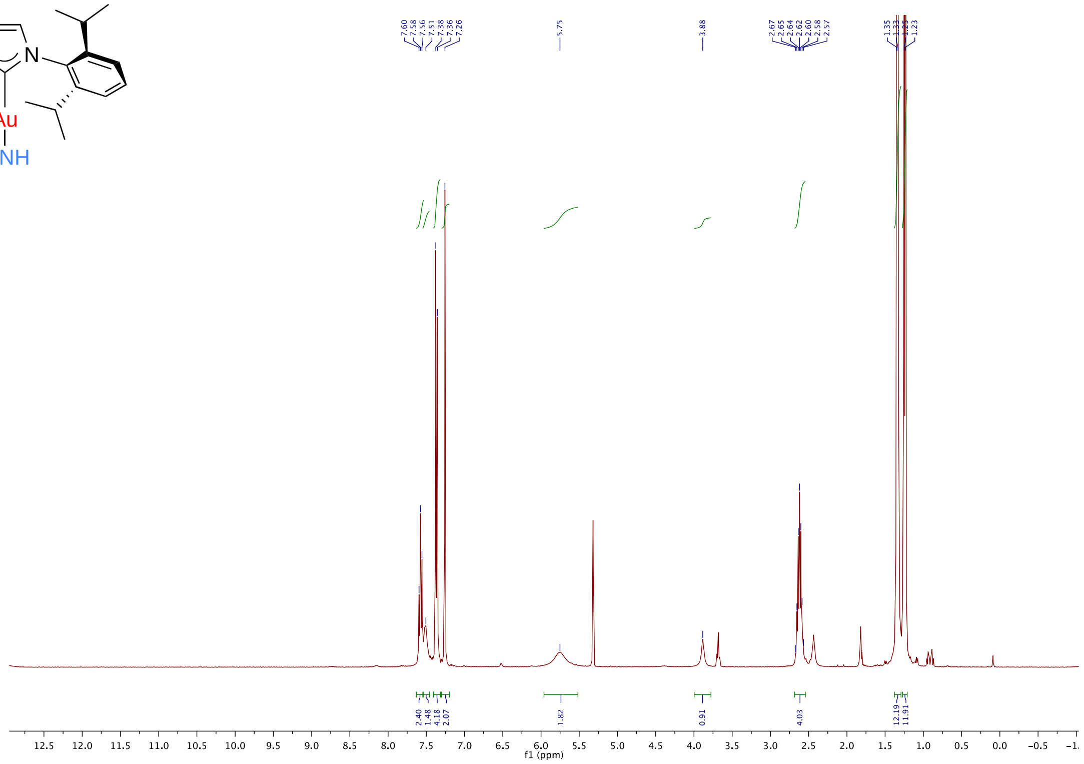
7.60
7.58
7.56
7.51
7.38
7.36
7.26
5.75
3.88
2.67
2.65
2.64
2.62
2.60
2.58
2.57
1.35
1.33
1.23
2.40
1.48
4.18
2.07
1.82
0.91
4.03
12.19
11.91
f1 (ppm)

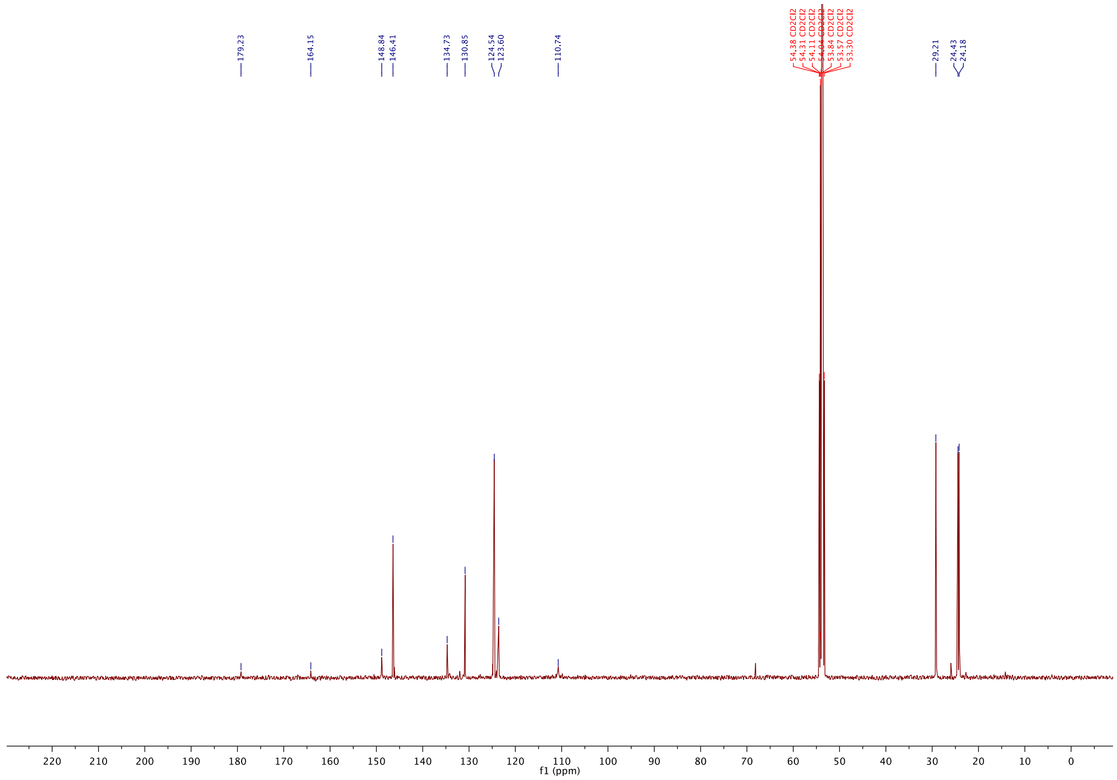
179.23
164.15
148.84
146.41
134.73
130.85
124.54
123.60
110.74
54.38 CD2Cl2
54.31 CD2Cl2
54.11 CD2Cl2
54.04 CD2Cl2
53.84 CD2Cl2
53.57 CD2Cl2
53.30 CD2Cl2
29.21
24.43
24.18
f1 (ppm)

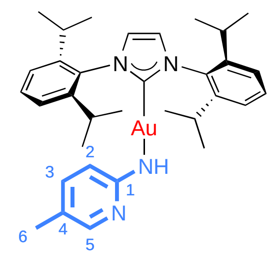
Au
NH
N
1
2
3
4
5
6

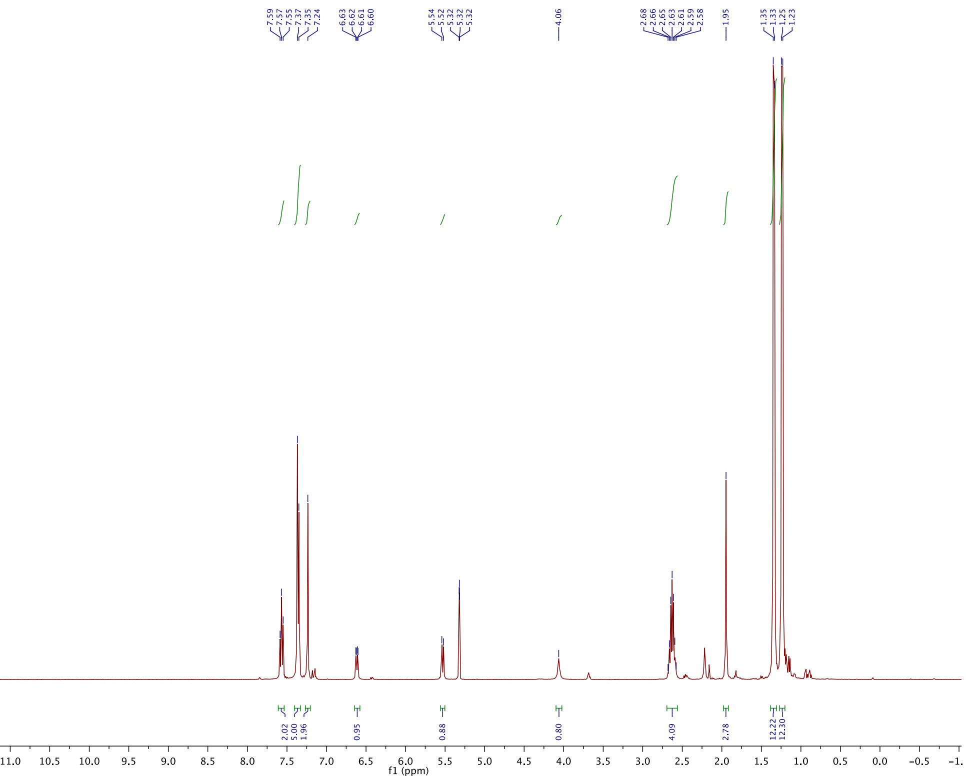
7.59
7.57
7.55
7.37
7.35
7.24
6.63
6.62
6.61
6.60
5.54
5.52
5.32
5.32
5.32
4.06
2.68
2.66
2.65
2.63
2.61
2.59
2.58
1.95
1.35
1.33
1.25
1.23
2.02
5.00
1.96
0.95
0.88
0.80
4.09
2.78
12.22
12.30
f1 (ppm)

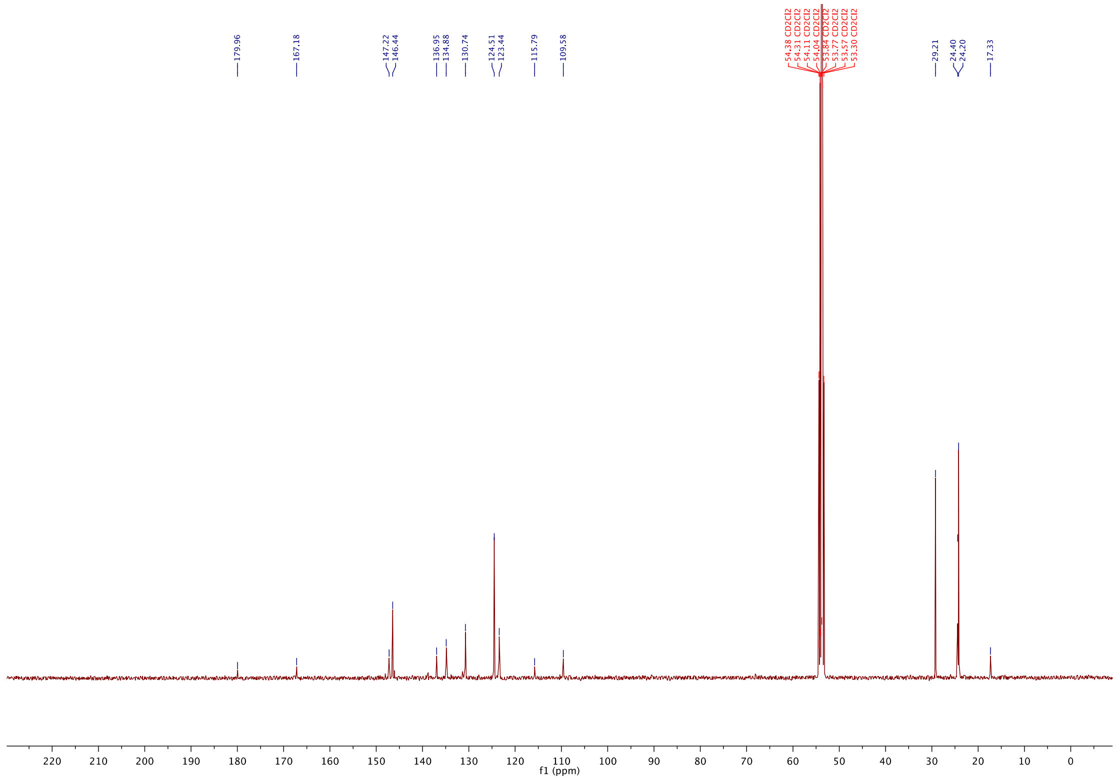
179.96
167.18
147.22
146.44
136.95
134.88
130.74
124.51
123.44
115.79
109.58
29.21
24.40
24.20
17.33
f1 (ppm)

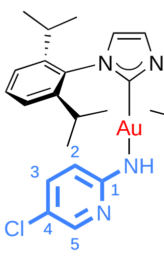
Au
NH
N
Cl
1
2
3
4
5

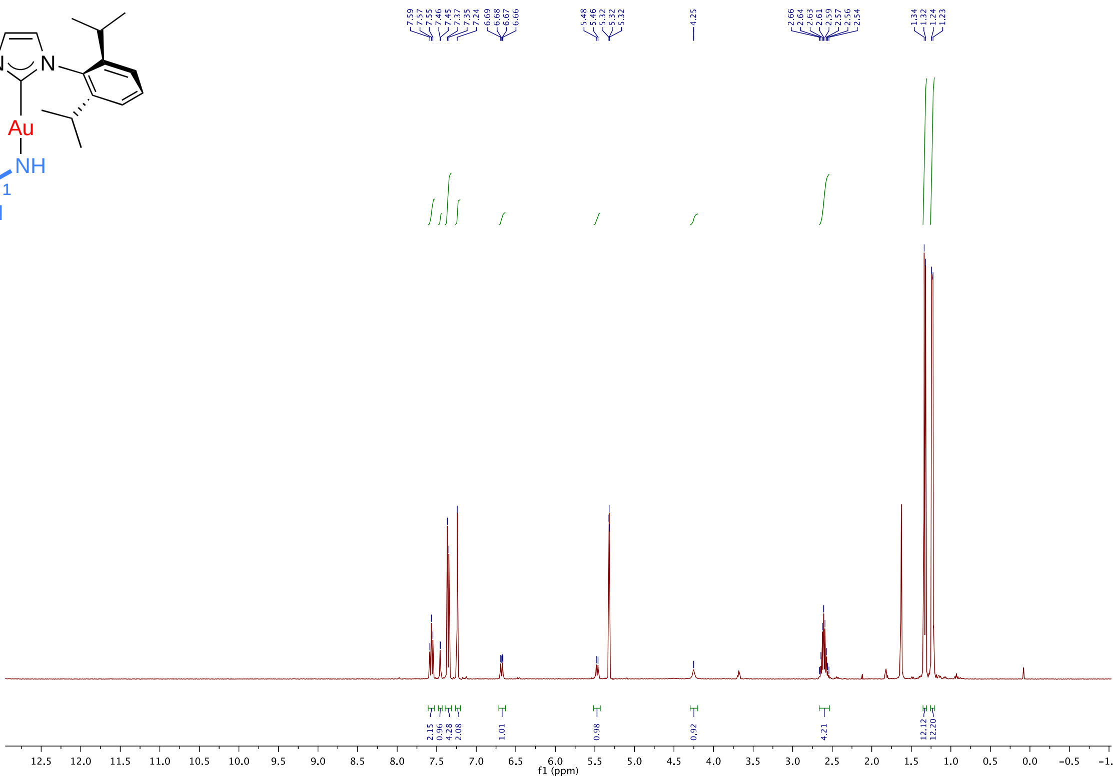
7.59
7.57
7.55
7.46
7.45
7.37
7.35
7.24
6.69
6.68
6.67
6.66
5.48
5.46
5.32
5.32
5.32
4.25
2.66
2.64
2.63
2.61
2.59
2.57
2.56
2.54
1.34
1.32
1.24
1.23
2.15
0.96
4.28
2.08
1.01
0.98
0.92
4.21
12.12
12.20
f1 (ppm)

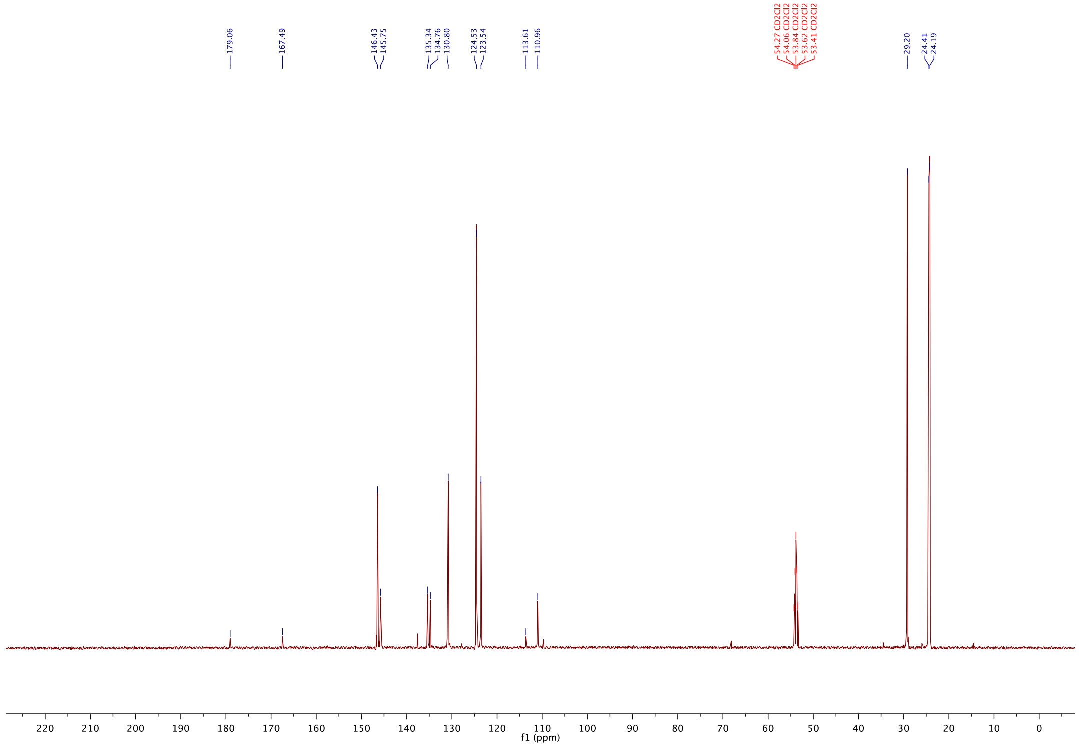
179.06
167.49
146.43
145.75
135.34
134.76
130.80
124.53
123.54
113.61
110.96
54.27 CD2Cl2
54.06 CD2Cl2
53.84 CD2Cl2
53.62 CD2Cl2
53.41 CD2Cl2
29.20
24.41
24.19
f1 (ppm)

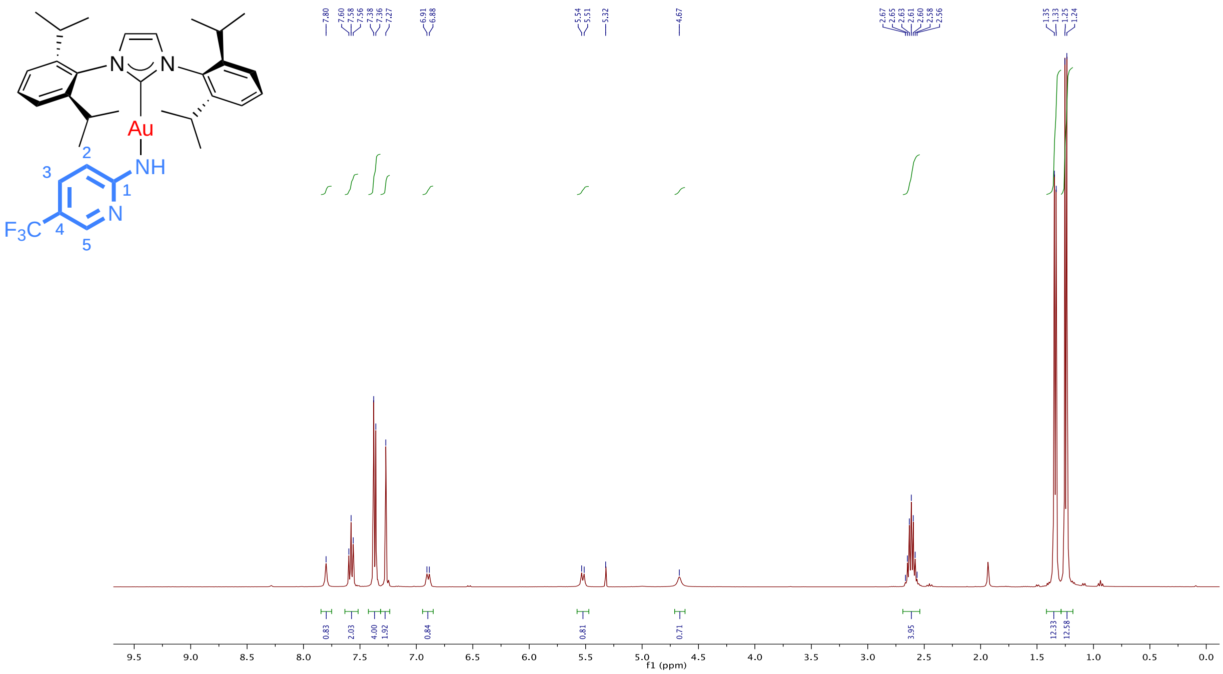
Au
NH
N
F3C
1
2
3
4
5
7.80
7.60
7.58
7.56
7.38
7.36
7.27
6.91
6.88
5.54
5.51
5.32
4.67
2.67
2.65
2.63
2.61
2.60
2.58
2.56
1.35
1.33
1.25
1.24
0.83
2.03
4.00
1.92
0.84
0.81
0.71
3.95
12.33
12.58
f1 (ppm)

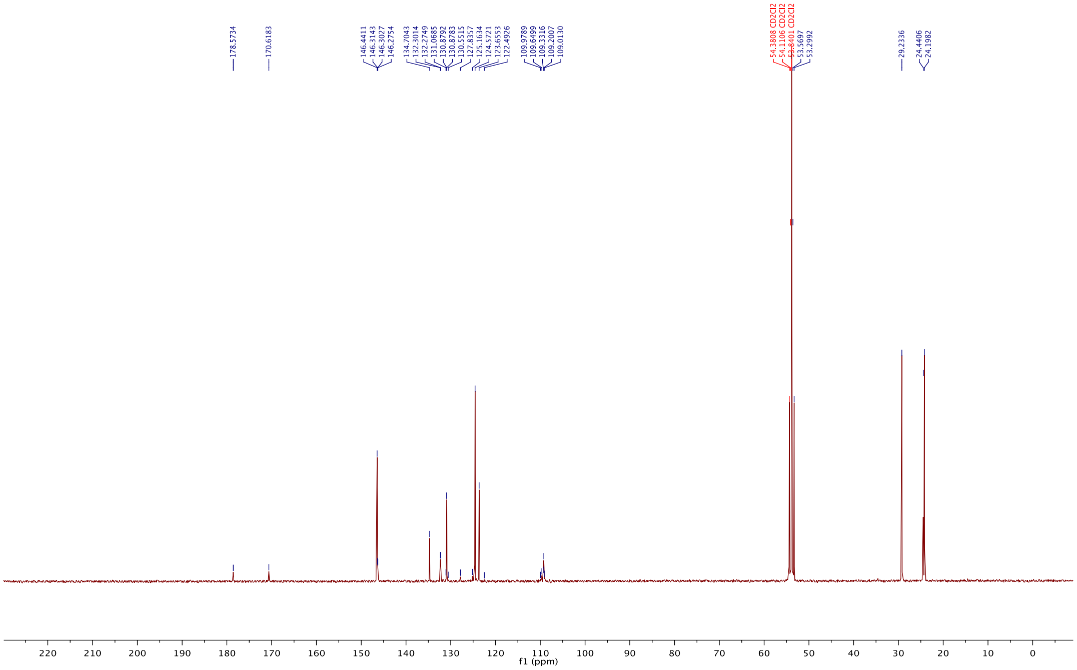
178.5734
170.6183
146.4411
146.3143
146.3027
146.2754
134.7043
132.3014
132.2749
131.0685
130.8792
130.8783
130.5515
127.8357
125.1634
124.5721
123.6553
122.4926
109.9789
109.6499
109.3316
109.2007
109.0130
54.3808 CD2Cl2
54.1106 CD2Cl2
53.8401 CD2Cl2
53.5697
53.2992
29.2336
24.4406
24.1982
f1 (ppm)

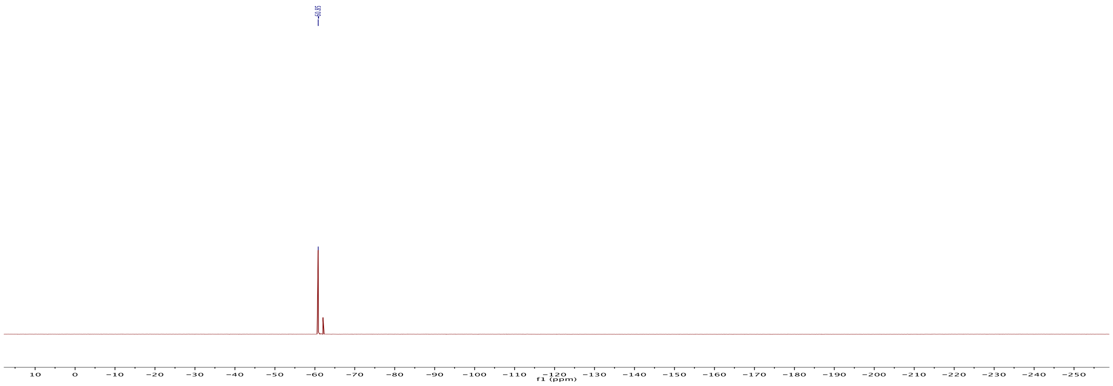
f1 (ppm)

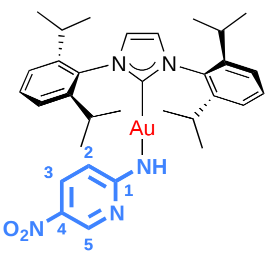
Au
NH
N
N
N
O2N
1
2
3
4
5

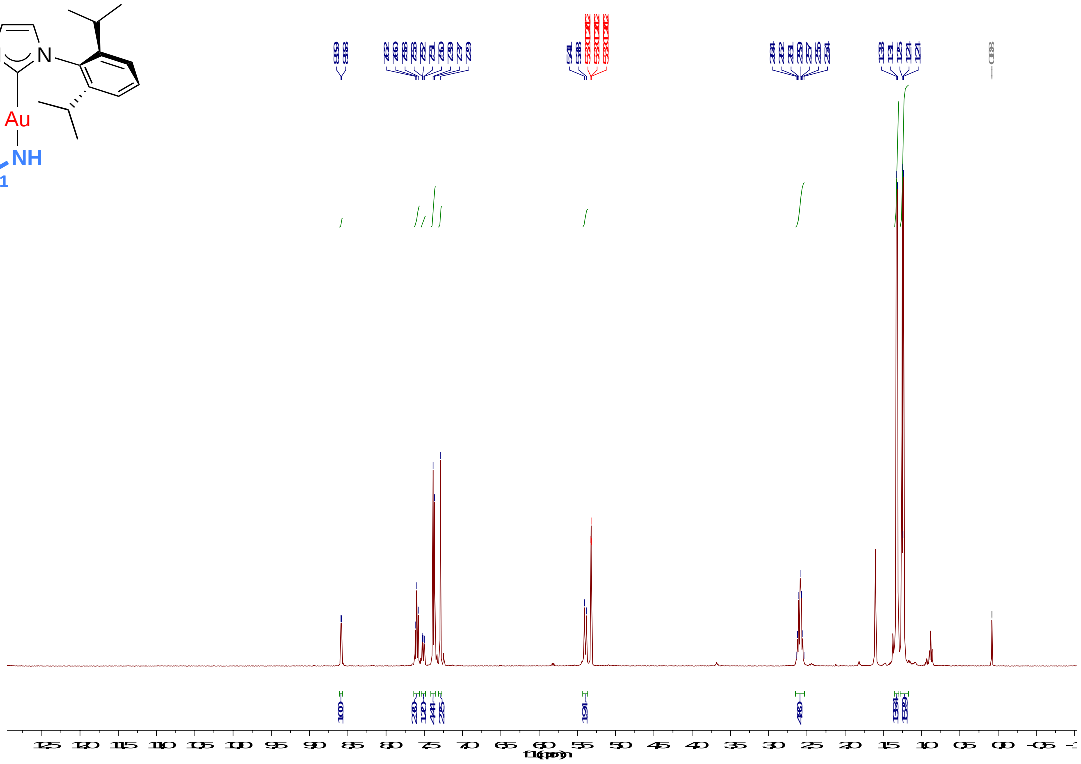

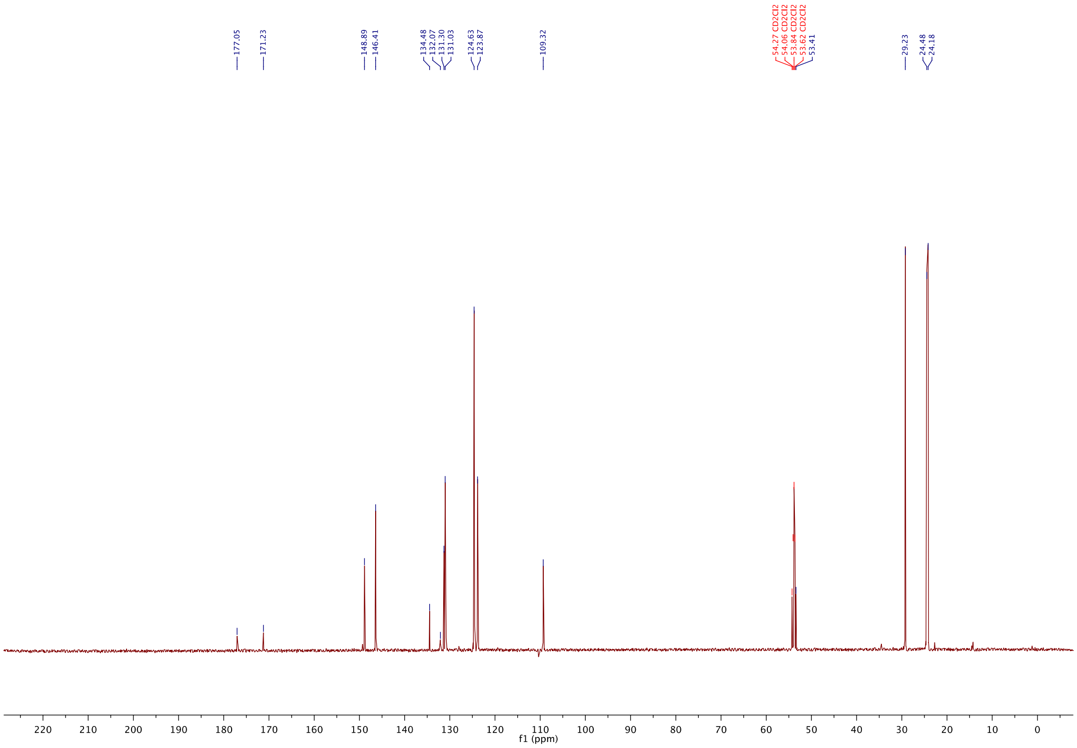
177.05
171.23
148.89
146.41
134.48
132.07
131.30
131.03
124.63
123.87
109.32
54.27 CD2Cl2
54.06 CD2Cl2
53.84 CD2Cl2
53.62 CD2Cl2
53.41
29.23
24.48
24.18
220
210
200
190
180
170
160
150
140
130
120
110
100
90
80
70
60
50
40
30
20
10
0
f1 (ppm)

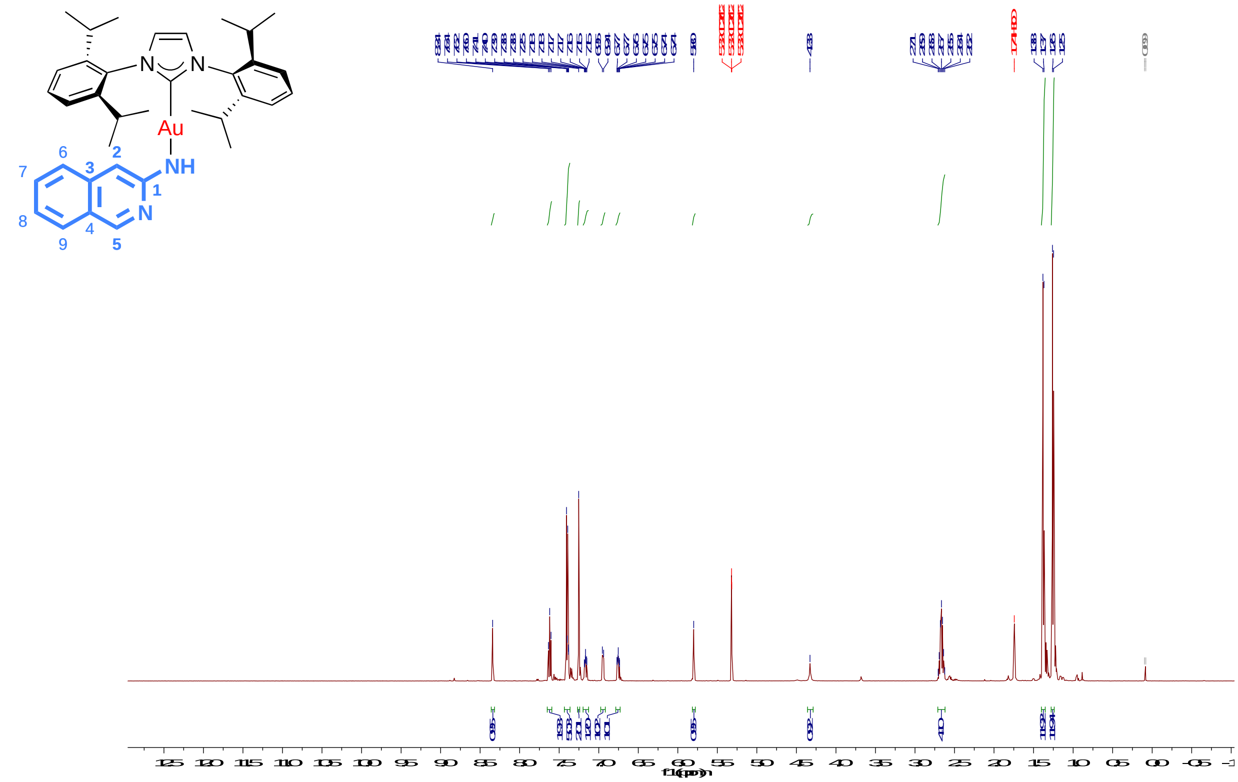
Au
NH
N
f1 (ppm)

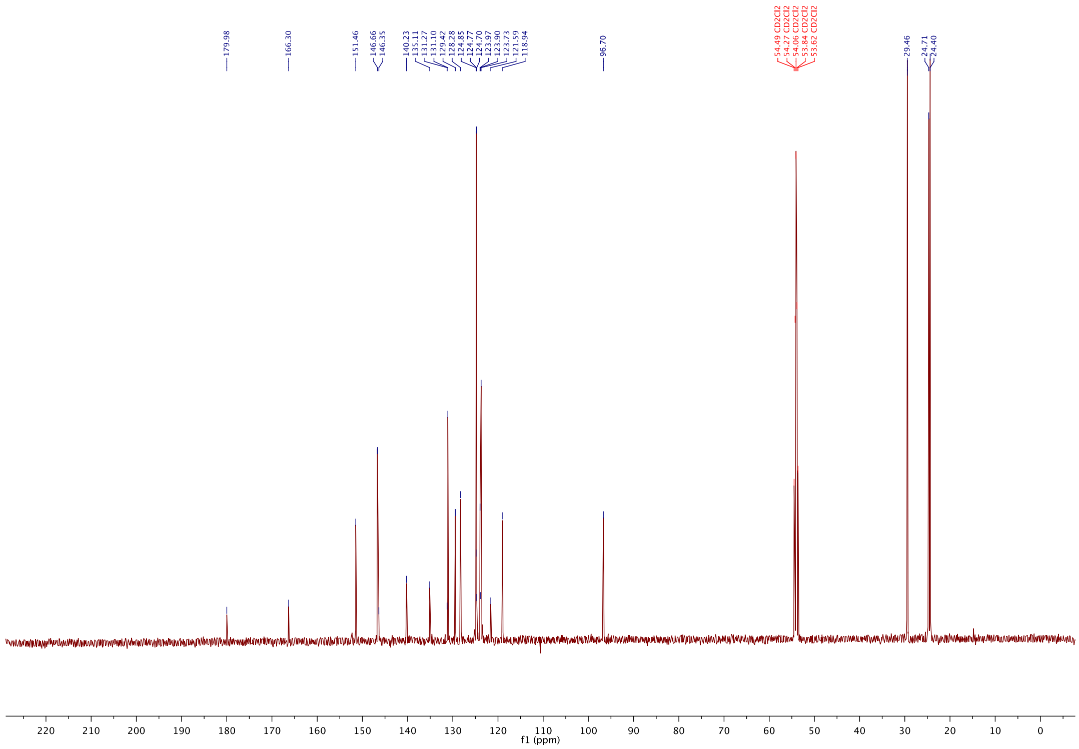
179.98
166.30
151.46
146.66
146.35
140.23
135.11
131.27
131.10
129.42
128.28
124.85
124.77
124.70
123.97
123.90
123.73
121.59
118.94
96.70
54.49 CD2Cl2
54.27 CD2Cl2
54.06 CD2Cl2
53.84 CD2Cl2
53.62 CD2Cl2
29.46
24.71
24.40
f1 (ppm)

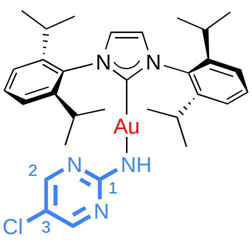
N
N
Au
NH
N
N
Cl
1
2
3

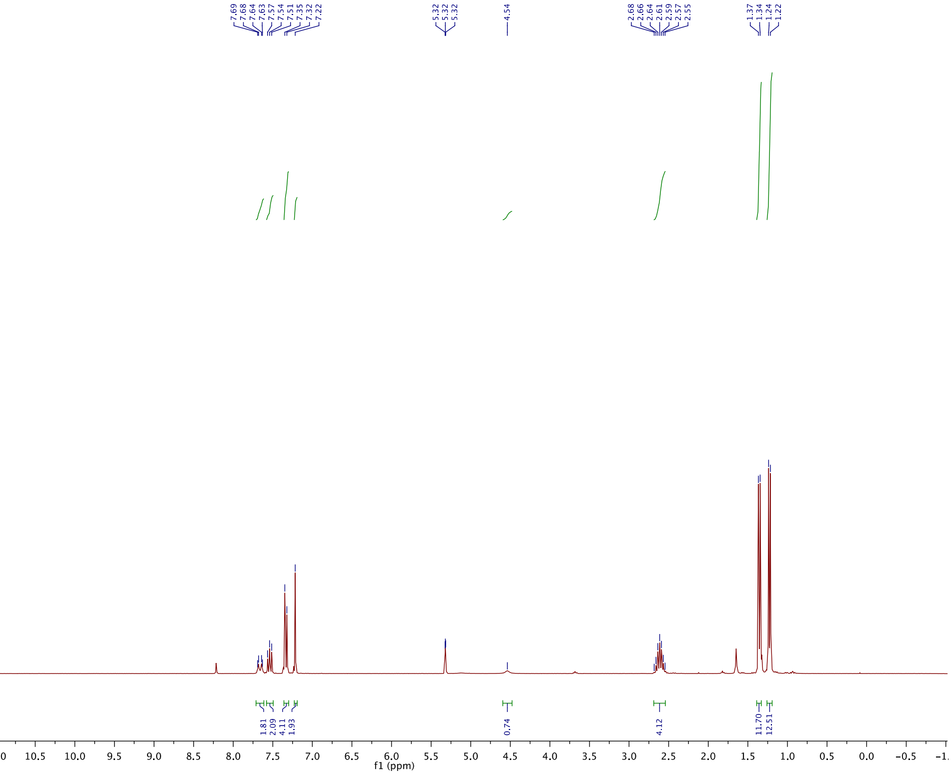
7.69
7.68
7.64
7.63
7.57
7.54
7.51
7.35
7.32
7.22
5.32
5.32
5.32
4.54
2.68
2.66
2.64
2.61
2.59
2.57
2.55
1.37
1.34
1.24
1.22
1.81
2.09
4.11
1.93
0.74
4.12
11.70
12.51
f1 (ppm)

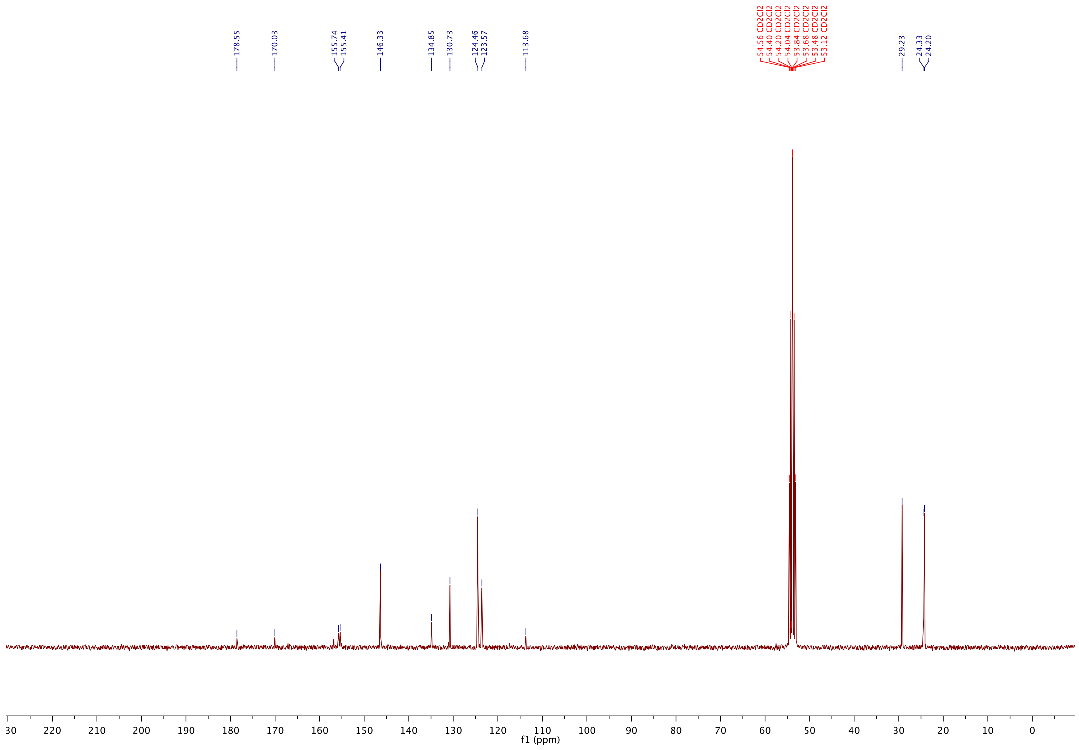
178.55
170.03
155.74
155.41
146.33
134.85
130.73
124.46
123.57
113.68
54.56 CD2Cl2
54.40 CD2Cl2
54.20 CD2Cl2
54.04 CD2Cl2
53.84 CD2Cl2
53.68 CD2Cl2
53.48 CD2Cl2
53.12 CD2Cl2
29.23
24.33
24.20
f1 (ppm)

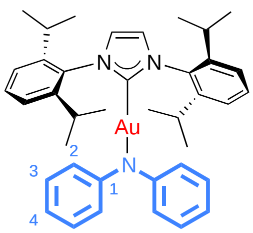

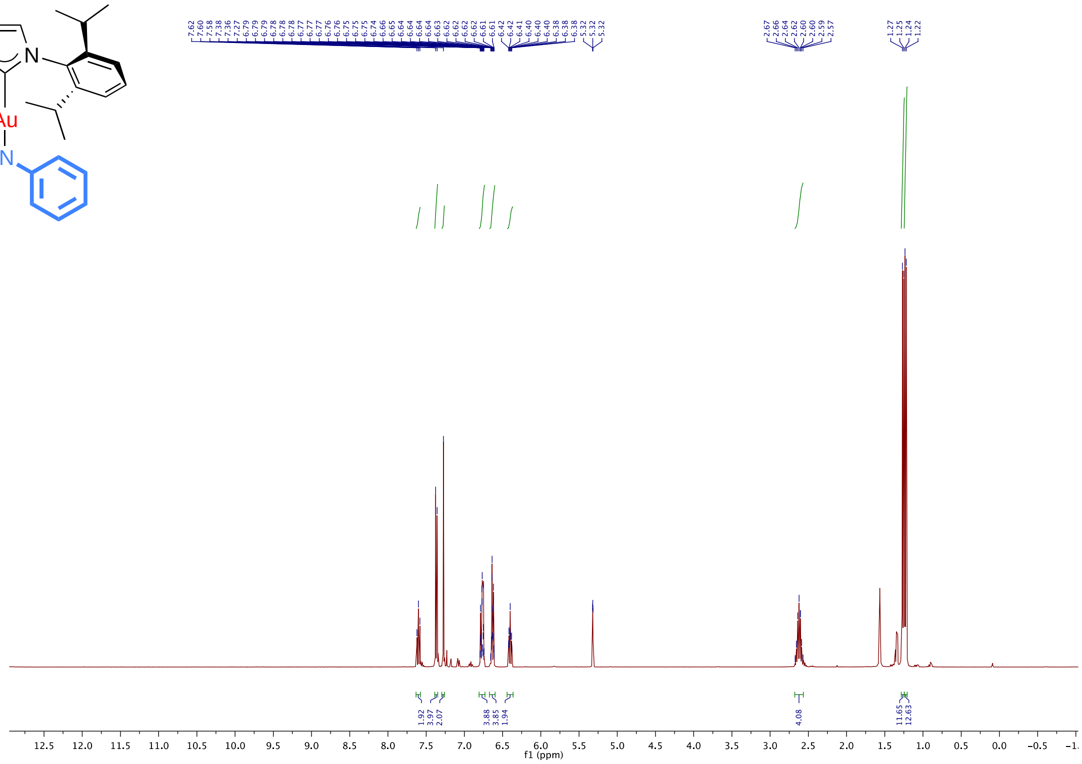

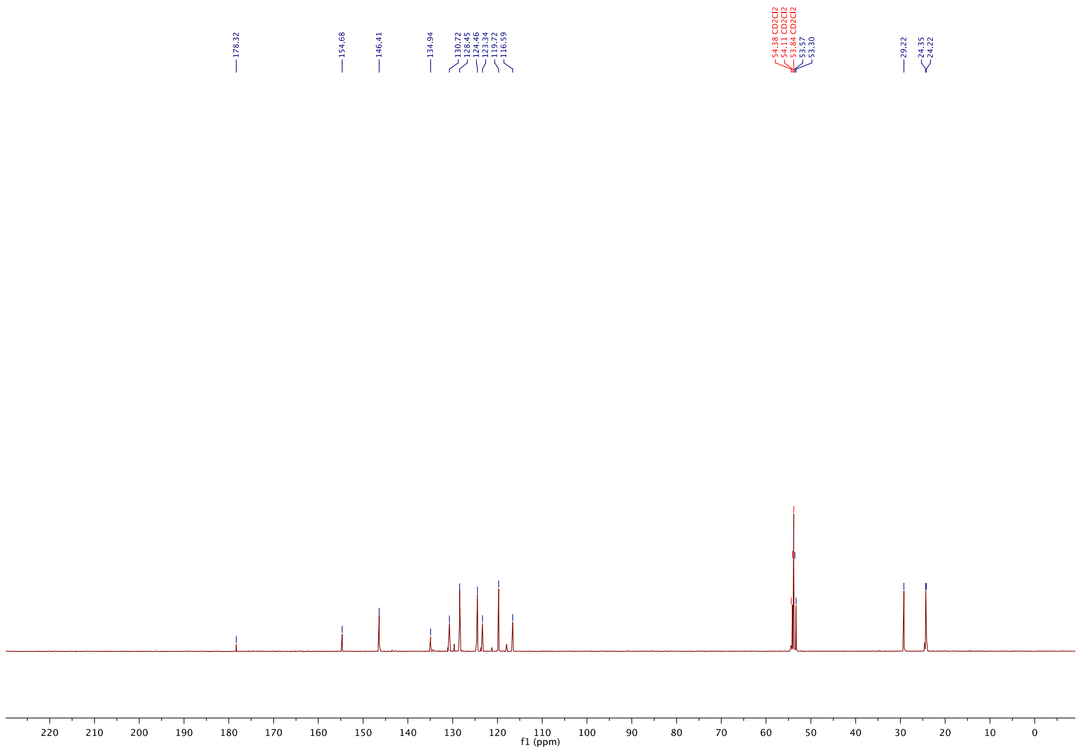

DFT Calculations

Calculations were performed at the M06-L/SDD level of theory using Gaussian 09.[1] The crystal structure of **3** was used as the starting point for optimization. The structure was then optimized using tight convergence criteria and the ultrafine grid. The resulting structure was confirmed to be a minimum *via* frequency calculations (no imaginary frequencies). The co-ordinates are listed below.

[1] M. J. Frisch, G. W. Trucks, H. B. Schlegel, G. E. Scuseria, M. A. Robb, J. R. Cheeseman, J. A. Montgomery, T. Vreven, K. N. Kudin, J. C. Burant, J. M. Millam, S. S. Iyengar, J. Tomasi, V. Barone, B. Mennucci, M. Cossi, G. Scalmani, N. Rega, G. A. Petersson, H. Nakatsuji, M. Hada, M. Ehara, K. Toyota, R. Fukuda, J. Hasegawa, M. Ishida, T. Nakajima, Y. Honda, O. Kitao, H. Nakai, M. Klene, X. Li, J. E. Knox, H. P. Hratchian, J. B. Cross, C. Adamo, J. Jaramillo, R. Gomperts, R. E. Stratmann, O. Yazyev, A. J. Austin, R. Cammi, C. Pomelli, J. W. Ochterski, P. Y. Ayala, K. Morokuma, G. A. Voth, P. Salvador, J. J. Dannenberg, V. G. Zakrzewski, S. Dapprich, A. D. Daniels, M. C. Strain, O. Farkas, D. K. Malick, A. D. Rabuck, K. Raghavachari, J. B. Foresman, J. V. Ortiz, Q. Cui, A. G. Baboul, S. Clifford, J. Cioslowski, B. B. Stefanov, G. Liu, A. Liashenko, P. Piskorz, I. Komaromi, R. L. Martin, D. J. Fox, T. Keith, M. A. Al-Laham, C. Y. Peng, A. Nanayakkara, M. Challacombe, P. M. W. Gill, B. Johnson, W. Chen, M. W. Wong, C. Gonzalez and J. A. Pople, Gaussian 03, Revision D.02, Gaussian Inc., Wallingford, CT, 2004.

3

Au	-0.16857	-0.98801	-0.29594	C	-3.39182	1.81755	1.72913
N	2.13052	0.98404	0.32002	C	-2.00188	1.60762	1.74446
N	0.16733	1.86979	0.61737	C	-1.07330	2.94213	-1.76896
N	-1.25030	-2.64014	-0.79584	C	-1.44379	4.29862	-2.38909
C	0.78224	0.69521	0.23236	C	-1.26969	1.79922	-2.78884
C	2.34169	2.30190	0.75297	C	-1.35315	0.89277	2.91924
C	1.10538	2.85697	0.94254	C	-1.55358	1.67306	4.23149
C	3.17595	0.04263	-0.01409	C	-1.90297	-0.54236	3.03518
C	3.86150	-0.59897	1.03944	C	-2.61505	-2.59537	-0.72309
C	4.88391	-1.49870	0.69103	C	-4.50406	-1.30611	-0.20774
C	5.18843	-1.75302	-0.65120	C	-5.39340	-2.34485	-0.51622
C	4.47220	-1.11911	-1.67366	C	-4.83743	-3.56651	-0.95031
C	3.44643	-0.20502	-1.37802	C	-3.45600	-3.70079	-1.05621
C	3.44189	-0.39619	2.48939	H	3.32997	2.70634	0.87982
C	4.60263	-0.54448	3.48324	H	0.80882	3.83840	1.26556
C	2.30260	-1.38065	2.83627	H	5.43438	-2.00926	1.47409
C	2.68156	0.51843	-2.47862	H	5.97729	-2.45377	-0.90111
C	3.32666	1.89058	-2.76668	H	4.70581	-1.33947	-2.70967
C	2.55737	-0.30521	-3.76711	H	3.03848	0.61932	2.59458
C	-1.26914	2.05059	0.62633	H	4.96615	-1.57695	3.52154
C	-1.87152	2.62993	-0.51086	H	4.26815	-0.28346	4.49114
C	-3.26341	2.81585	-0.47974	H	5.44584	0.10214	3.22255
C	-4.01495	2.42621	0.63538	H	2.65944	-2.41416	2.76788

H	1.45508	-1.27512	2.14838
H	1.94405	-1.20797	3.85669
H	1.66265	0.69821	-2.11540
H	2.75012	2.43109	-3.52467
H	4.34960	1.76651	-3.13938
H	3.36684	2.51213	-1.86579
H	3.52594	-0.43519	-4.26307
H	1.89589	0.20648	-4.47241
H	2.13671	-1.29409	-3.56461
H	-3.76189	3.25186	-1.34006
H	-5.08900	2.57777	0.64101
H	-3.98634	1.48355	2.57487
H	-0.00644	2.97351	-1.51122
H	-2.48035	4.30542	-2.74244
H	-0.80436	4.50685	-3.25224
H	-1.32830	5.11517	-1.66994
H	-2.31808	1.73778	-3.10051
H	-0.99966	0.82916	-2.35342
H	-0.65394	1.96836	-3.67978
H	-0.27438	0.81927	2.73281
H	-1.06671	1.15313	5.06246
H	-2.61825	1.76485	4.47366
H	-1.13654	2.68339	4.16524
H	-2.97400	-0.53346	3.26972
H	-1.38405	-1.08696	3.83110
H	-1.77986	-1.08495	2.09111
H	-0.86078	-3.52560	-1.10092
H	-4.87632	-0.34180	0.13202
H	-6.46333	-2.20768	-0.42292
H	-5.48131	-4.40313	-1.20216
H	-3.00866	-4.63299	-1.38768
N	-3.15946	-1.39987	-0.29950
